# Insights into Peptidyl-Prolyl *cis*-*trans* Isomerases from Clinically Important Protozoans: From Structure to Potential Biotechnological Applications

**DOI:** 10.3390/pathogens13080644

**Published:** 2024-07-31

**Authors:** Verónica Aranda-Chan, Rosa Elena Cárdenas-Guerra, Alejandro Otero-Pedraza, Esdras Enoc Pacindo-Cabrales, Claudia Ivonne Flores-Pucheta, Octavio Montes-Flores, Rossana Arroyo, Jaime Ortega-López

**Affiliations:** 1 Departamento de Biotecnología y Bioingeniería, Centro de Investigación y de Estudios Avanzados del Instituto Politécnico Nacional (CINVESTAV-IPN), Av. IPN # 2508, Col. San Pedro Zacatenco, Gustavo A. Madero, Mexico City 07360, Mexico; veronica.aranda@cinvestav.mx (V.A.-C.); janeiro2606@gmail.com (R.E.C.-G.); otero_3333@hotmail.com (A.O.-P.); esdras.pacindo@cinvestav.mx (E.E.P.-C.); cflores@cinvestav.mx (C.I.F.-P.); red_bull133@hotmail.com (O.M.-F.); 2 Departamento de Infectómica y Patogénesis Molecular, Centro de Investigación y de Estudios Avanzados del Instituto Politécnico Nacional (CINVESTAV-IPN), Av. IPN # 2508, Col. San Pedro Zacatenco, Gustavo A. Madero, Mexico City 07360, Mexico; rarroyo@cinvestav.mx

**Keywords:** PPIases, protozoan parasites, host–pathogen interactions, pathogenesis, recombinant proteins, vaccines

## Abstract

Peptidyl-prolyl *cis*/*trans* isomerases (PPIases) are present in a wide variety of microorganisms, including protozoan parasites such as *Trypanosoma cruzi*, *Trypanosoma brucei*, *Trichomonas vaginalis*, *Leishmania major*, *Leishmania donovani*, *Plasmodium falciparum*, *Plasmodium vivax*, *Entamoeba histolytica*, *Giardia intestinalis*, *Cryptosporidium parvum*, and *Cryptosporidium hominis*, all of which cause important neglected diseases. PPIases are classified as cyclophilins, FKBPs, or parvulins and play crucial roles in catalyzing the *cis-trans* isomerization of the peptide bond preceding a proline residue. This activity assists in correct protein folding. However, experimentally, the biological structure–function characterization of PPIases from these protozoan parasites has been poorly addressed. The recombinant production of these enzymes is highly relevant for this ongoing research. Thus, this review explores the structural diversity, functions, recombinant production, activity, and inhibition of protozoan PPIases. We also highlight their potential as biotechnological tools for the in vitro refolding of other recombinant proteins from these parasites. These applications are invaluable for the development of diagnostic and therapeutic tools.

## 1. Introduction

Parasitic protozoa are responsible for a significant number of diseases worldwide, leading to an estimated of 500 million people affected by morbidity and dead annually [1]. Owing to their relevance in public health, numerous parasite genomes have been partially or entirely described to better understand the genes and proteins associated with these organisms [2].

Peptidyl-prolyl cis/trans isomerases (PPIases) are specialized enzymes that catalyze conformational changes in proteins and are highly conserved in all living organisms, including archaea, bacteria, protists, and eukaryotes. They participate in various biological processes, such as protein folding, protein trafficking, cell signaling, and immune response, and they can act as virulence factors [3,4,5].

The overall domain architecture of human PPIases (hPPIases) shows remarkable similarity to that in other organisms. hPPIase orthologs have been found in clinically important protozoan parasites such as *Trichomonas*, *Entamoeba*, *Giardia*, *Trypanosoma*, *Leishmania*, *Plasmodium*, *Toxoplasma*, and *Cryptosporidium* species, emphasizing their importance in the survival, development, and pathogenicity of these organisms [6]. However, the role of PPIases in parasites remains unknown.

**Figure 1:**
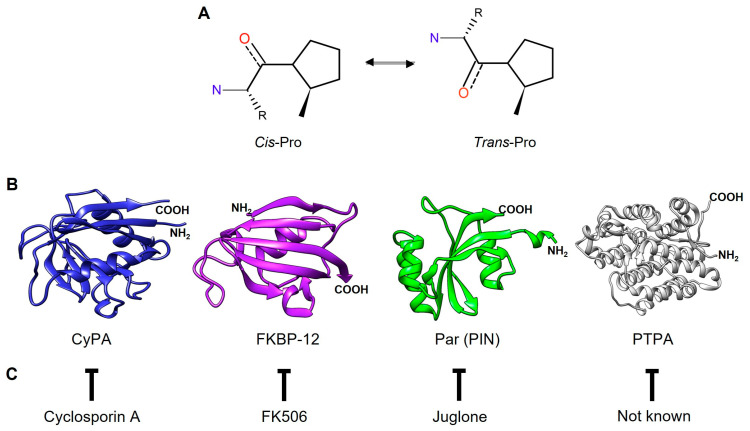
Families of PPIase proteins. (**A**) PPIase activity: *cis-trans* isomerization of X-Pro bonds. (**B**) Crystal structures of human PPIases from the four different families: cyclophilins (CyP), FK506-binding proteins (FKBP), parvulins (Par), and protein phosphatase two A phosphatase activator (PTPA). Cyclophilin A is indicated in dark blue (PDB: 3K0M). FKBP12 (PDB: 2PPN). PIN1 is indicated in green (PDB 1PIN). PTPA (PDB 2IXM) is indicted in gray. (**C**) Inhibitors of different PPIase families.

PPIases facilitate the interconversion of the *cis*- and *trans*-isomers of the N-terminal bond preceding a proline residue (X-Pro) in nascent proteins (Figure 1A). The *trans* conformation is generally more favorable for most amino acids (aa), except for proline, where the difference in free energy between the *cis* and *trans* conformations is small. Furthermore, most *cis* prolines are exposed on the protein surface [7]. Although the exact mechanism of action of PPIases has not been determined, several theories, including substrate desolvation, substrate autocatalysis, preferential transition-state binding, and nucleophilic catalysis, have been proposed. The proper conformation of these bonds in a protein is crucial, since different functions can rely on the distinction between the *cis* and *trans* states [5].

The superfamily of PPIases includes four families of nonhomologous proteins, namely cyclophilins (CyPs), FK506-binding proteins (FKBPs), parvulins (Pars), and protein phosphatase 2A phosphatase activators (PTPAs). Each family can be distinguished by specific inhibitors (Figure 1C). For instance, CyPs and FKBPs are inhibited by immunosuppressive drugs such as cyclosporin A (CsA) and FK506 (tacrolimus), respectively. FKBPs can also be inhibited by rapamycin (sirolimus). Pars, on the other hand, are inhibited by the natural compound juglone (5-hydroxy-1,4-naphthalenedione). However, specific PTPA inhibitors have not yet been identified [4,8,9,10]. PPIases are characterized by a catalytic domain located in the protein’s central region responsible for the PPIase activity. Additionally, these enzymes have isoforms with sequence extensions in either the N- or C-terminal region or in both regions, and these isoforms are associated with their specific cellular functions.

The discovery that mammalian tissues contain CyPs with a high affinity for CsA marked the beginning of PPIase research [3,11]. Human CyPs (hCyPs) have been extensively studied. The structure of CyPs is a closed, eight-stranded, antiparallel β-barrel structure with two α helixes enclosing the barrel from either side (Figure 1B). These proteins share a common domain of ~109 aa known as the cyclophilin-like domain (CLD) [12]. Four hCyPs have been identified based on their localization to specific organelles; hCyP19 is linked to the nuclear spliceosome, CyPB is found in the endoplasmic reticulum (ER), CyPC is present in the membrane and has a signal peptide (SP) for the ER, and CyPD is associated with the mitochondria because an SP directs it to this organelle [12,13]. Furthermore, hCyPs share high sequence homology within their own family, and their active region contains highly conserved aa residues, such as R55, F60, M61, Q63, A101, F113, W121, L122, and H126 [14].

FKBPs contain an FKBP domain that is responsible for PPIase catalytic activity. hFKBP12 is a well-studied reference protein in this family. Structurally, FKBPs comprise a five-stranded β-sheet wrapped around one short α-helix, forming a β-barrel similar to that of CyPs (Figure 1B) [15,16,17]. These proteins are regarded as divergent due to their lack of universally conserved aa residues, which underlies their unique and varied nature [17].

Pars, the third type of enzymes with PPIase activity, are structurally unrelated to CyPs and FKBPs. The term “parvulins” is derived from the Latin word “parvulus”, meaning “very small”, and refers to the low molecular weight of these proteins [18,19,20]. hPar (Pin1) is a well-characterized nuclear protein, and its tertiary structure contains four β-strands, one α-helix, and a loop. Pin1 consists of the following two domains: (1) the N-terminal WW domain, which is a protein–protein interaction motif related to its cellular localization through recognition of proline-rich peptide motifs (PRMs) and phosphorylated phosphate Ser/Thr-Pro sites [21], and (2) the C-terminal catalytic domain (PpiC), which promotes the isomerization of the Ser-Pro or Thr-Pro bonds [22,23] (Figure 1B).

In addition to the extensive investigations of hPPIases, additional research is needed to understand the role of PPIases in parasites. This work provides an overview of the current information on PPIases reported in databases (VEuPathDB and UniProt) of clinically relevant protozoan parasites, such as *Trichomonas vaginalis*, *Entamoeba histolytica*, *Giardia intestinalis*, *Trypanosoma cruzi*, *Trypanosoma brucei*, *Leishmania major*, *Leishmania donovani*, *Plasmodium falciparum*, *Plasmodium vivax*, *Toxoplasma gondii*, *Cryptosporidium parvum*, and *Cryptosporidium hominis*. We focus on the structural characteristics, localization, and functions of these proteins. Similarly, we analyze several biotechnological aspects. We collected important information about the expression processes, purification, and activity of recombinantly produced protozoan PPIases, and we discuss the possible biotechnological applications of these proteins in assisted protein refolding.

## 2. Parasite PPIases: Disease, Genome Database, and Structural Characteristics

### 2.1. Anaerobic or Microaerophilic Protozoan Parasites

#### 2.1.1. *Trichomonas vaginalis*

Trichomoniasis is caused by protist parasite *T. vaginalis*, considered the most common non-viral sexually transmitted disease worldwide. In the USA, an estimated 2.6 million infected people, with a prevalence of 2.1% in women and 0.5% in men, have been recently reported. Infected people can present a variety of symptoms, but most are asymptomatic. Trichomonal infection has been linked to other sexually transmitted diseases, such as HIV, and ~53% of women who have HIV also have *T. vaginalis.* Infection with this parasite is also considered a risk factor for vertical transmission of HIV [24].

The genome of *T. vaginalis* strain G3 encodes 13 CyPs, 9 FKBPs, and 3 Pars, as reported in the TrichDB database (https://trichdb.org/trichdb/app/, accessed on 1 June 2023) (Release 63 3 May 2023) [25] (Table 1). In this review, we use the nomenclature for PPIases reported by Galat (2003) [13], in which PPIases are named with a prefix of two letters indicating the species, followed by a number for the calculated molecular weight in kDa.

The PPIases from *T. vaginalis* present sequence variations within each family, with few conserved regions. Phylogenetic analysis revealed that the CyPs were grouped into clades with low bootstrap values, suggesting that they do not share a common origin. In contrast, the FKBPs and Pars formed distinct clades with higher bootstrap values, indicating a common evolutionary origin for these proteins. However, TvCyP44 was found to share a clade with the FKBPs, which might indicate that it is more closely related to FKBPs than to CyPs, despite containing a CLD (Supplementary Figure S1, [26]).

Most CyPs from *T. vaginalis* retain the following aa residues essential for catalytic activity within the highly conserved CLD identified in TvCyP19 (referred to as TvCyP1 by Hsu et al., 2014) [27]: H^62^, R^63^, F^68^, M^69^, Q^71^, G^80^, A^109^, N^110^, A^111^, Q^119^, F^121^, W^129^, L^130^, and H^134^. Exceptions to this pattern were observed in TvCyP21, TvCyP37, and TvCyP44 (Supplementary Figure S2, [25,28]).

**Table 1:** Peptidyl-prolyl cis-trans isomerase repertoire from *Trichomonas vaginalis* ^1^.

UniProt	TrichDB ^2^	TrichDB ^3^	NCBI	PDB	PPIase Name	Localization ^4^	Function ^4^	References
A2FJP1	TVAG_370440	TVAGG3_0054050	XP_001307803		TvCyP14	Nucleus		[29]
A2EC21	TVAG_137880	TVAGG3_0269460	XP_001322019.1		TvCyP18	Cytoplasm		[29]
A2DT06	TVAG_004440	TVAGG3_0649370	XP_001328636.1	5YB9	TvCyP19 (TvCyP1)	Cytoplasm hydrogenosomes, cytoplasm, and membrane	Protein trafficking	[30,31]
A2F1H0	TVAG_027250	TVAGG3_0947870	XP_001314072		TvCyP19.2	Cytoplasm		[29]
A2FAA8	TVAG_047830	TVAGG3_0485720	XP_001311112		TvCyP19.8	Cytoplasm		[29]
A2DLL4	TVAG_062520	TVAGG3_0580400	XP_001579633.1	6LXO	TvCyP19.9 TvCyP2	Cytoplasm ER, cytoplasm, and membrane	Protein trafficking	[32,33]
A2E5J4	TVAG_038810	TVAGG3_0240350	XP_001324258		TvCyP20	Cytoplasm		[29]
A2FIV3	TVAG_078570	TVAGG3_0462310	XP_001308098		TvCyP21	Cytoplasm		[29]
A2DKZ9	TVAG_146960	TVAGG3_0362200	XP_001579938		TvCyP22	Cytoplasm		[29]
A2FTU8	TVAG_27739	TVAGG3_0951420	XP_001304599		TvCyP23	Cytoplasm		[29]
A2E6H3	TVAG_106810	TVAGG3_0040330	XP_001324009.1		TvCyP37	Nucleus		[29]
A2GDG2	TVAG_583670	TVAGG3_0820230	XP_001297735.1		TvCyP44	Cytoplasm and nucleus		[29]
A2DEW6	TVAG_172150	TVAGG3_0530670	XP_001581944.1		TvCyP63	Nucleus		[29]
A2DA37	TVAG_476140	TVAGG3_0266130	XP_001583671.1		TvFKBP12	Cytoplasm		[29]
A2DYS7	TVAG_426610	TVAGG3_0538360	XP_001326690.1		TvFKBP15.1	ER		[29]
A2G763	TVAG_062070	TVAGG3_0922950	XP_001299933.1		TvFKBP15.2	ER		[29]
A2FYT1	TVAG_435000	TVAGG3_0194750	XP_001302863.1		TvFKBP19	Cytoplasm		[29]
A2F0D0	TVAG_292580	TVAGG3_0216440	XP_001330357.1		TvFKBP20	Cytoplasm		[29]
A2EV02	TVAG_368970	TVAGG3_0441630	XP_001315748.1		TvFKBP30	Cytoplasm		[29]
A2EC50	TVAG_413760	TVAGG3_0204900	XP_001321950.1		TvFKBP32	Cytoplasm		[29]
A2G9L9	TVAG_428320	TVAGG3_0107870	XP_001299079.1		TvFKBP33	Cytoplasm		[29]
A2FER9	TVAG_140950	TVAGG3_0603860	XP_001309536.1		TvFKBP63			[29]
A2ECU0	TVAG_102340	TVAGG3_0563910	XP_001321708.1		TvPar17.84	Cytoplasm		[29]
A2ED59	TVAG_420360	TVAGG3_0425040	XP_001321637.1		TvPar17.87	Cytoplasm and nucleus		[29]
A2EWG2	TVAG_325610	TVAGG3_0877000	XP_001315212.1		TvPar102	Cytoplasm and nucleus		[29]

^1^ Previously reported PPIase names are presented in parentheses. ^2^ The TrichDB database corresponds to TVAG of the G3 non-reference strain, the first classification. ^3^ The TrichDB database corresponds to TVAG and is associated with the genome update of the G3 2022 reference strain and a new classification. ^4^ The localization and function of PPIases were taken from the cited references or from the UniProt database and were predicted by the Gene Ontology Consortium [29]. ER: endoplasmic reticulum. Blank spaces: data not reported.

The structures of only two of the CyPs of *T. vaginalis* have been solved, namely those of TvCyP19 and TvCyP19.9 (referred to as TvCyP1 and TvCyP2, respectively, by Hsu et al., 2014; 2020) [27,30]. Both proteins present the typical structure, characterized by a β-barrel composed of eight antiparallel β-strands and two α-helixes. TvCyP19 mainly consists of the CLD domain, whereas TvC19.9, in addition to the catalytic domain, has a longer N-terminal segment. An important difference between the two CyPs is that TvCyP19 is a dimer, whereas TvCyP19.9 is a monomer [31,32].

**Figure 2:**
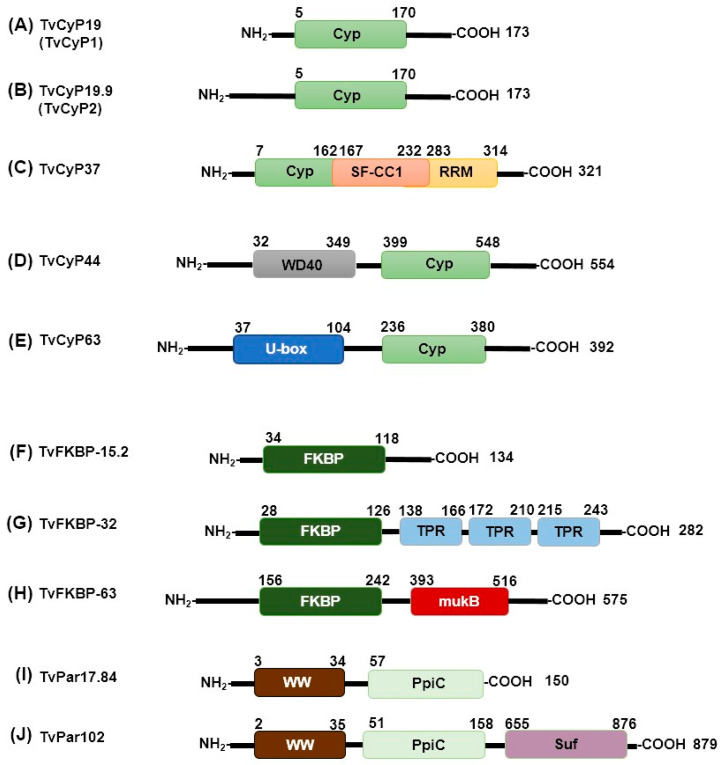
Domains present in PPIases of *T. vaginalis*. Examples of domains found in each family of *T. vaginalis* PPIases. (**A**–**E**), Cyclophilins (Cyp); (**F**–**H**); FKBP (**I**,**J**), parvulin (PpiC)-type PPIases. SF-CC1: splicing factor, CC1-like family; RRM: RNA recognition motif; WD-40: Trp-Asp dipeptide repeats; U-box: modified RING finger domain; TPR: Tetratricopeptide repeat; MukB: domain of chromosome partition protein MukB; WW: WWP repeating motif; Suf: Suppressor of forked domain. Numbers in bold indicate the length of the aa sequence. Sequences were retrieved from the UniProt database (https://www.uniprot.org/, accessed on 1 July 2023, Release 2023_02).

We analyzed the sequence identities of CyPs from *T. vaginalis,* taking the sequence of TvCyP19 as a reference (Supplementary Table S1, [28,34]). Low-molecular-weight CyPs (TvCyP14, TvCyP18, TvCyP19.2, TvCyP19.8, TvCyP20, TvCyP21, TvCyP22, and TvCyP23) have sequence identities of approximately 40–70% (Supplementary Table S1) with respect to TvCyP19. The eight small TvCyPs are characterized by having only the CLD in their sequence, similar to TvCyP19 and TvCyP19.9 (Figure 2A,B). Moreover, the high-molecular-weight CyPs (TvCyP37, TvCyP44, and TvCyP63) have low sequence identity to TvCyP19 (Supplementary Table S1). These TvCyPs exhibit a unique domain in addition to the conserved CLD. For example, TvCyP37 contains the SF-CC1 domain and the RRM motif at its C terminus (Figure 2C). The SF-CC1 domain is characteristic of RNA splicing factors and is marked by Arg- and Ser-rich sequences, typically followed by RNA recognition domains; this feature is also found in TvCyP37 [33]. Similarly, the presence of an RNA splicing factor has been noted in CyPs from other organisms, such as humans and *Arabidopsis thaliana* [35], along with the RNA recognition motif (RRM), which is involved in nucleic acid and/or protein recognition. The structural versatility of RRM interactions contributes to the diverse biological functions of RRM-containing proteins [36].

TvCyP44 has an N-terminal U-box domain, a specialized type of RING finger that differs from other RING fingers in terms of the lack of metal binding sites [37]. This domain has an estimated length of 67 aa (Figure 2D) and has been identified in ubiquitin ligase-like proteins in *Saccharomyces cerevisiae* that serve as scaffolds for proteins during ubiquitination and is associated with protein degradation pathways [38,39]. TvCyP63 contains a WD40 domain near the N terminus (aa 32–349; Figure 2E). This WD40 domain is also present in other large CyPs, such as PfCyP87, PvCyP83, and TgCyP86 from *P. falciparum*, *P. vivax*, and *T. gondii,* respectively. The WD40 domains function in anchoring to other proteins or in DNA binding. These domains are present in a wide variety of proteins with diverse functions, including chaperone proteins, but have no catalytic activity [40].

Compared to CyPs, *T. vaginalis* FKBPs presented lower sequence identities (less than 24%) when TvFKBP-12 was used as a reference (Supplementary Table S1). This low similarity is attributed to the absence of consensus sequences in FKBPs. Each of the nine TvFKBPs has an FKBP domain with a sequence length in the range of ~84 to 100 aa near the N terminus (Figure 2F–H). Six of the FKBPs (TvFKBP-12, TvFKBP-15.1, TvFKBP-15.2, TvFKBP-19, TvFKBP-20, and TvFKBP-33) range from 12 to 33 kDa and consist of a single FKBP domain that spans most of the protein sequence (Figure 2F). In contrast, three of the FKBPs (TvFKBP-30, TvFKBP-32, and TvFKBP-63) include another domain in addition to the catalytic domain. TvFKBP-30 and TvFKBP-32 contain three tetratricopeptide repeat (TPR) motifs near the C terminus (Figure 2G). TPRs are structural motifs that usually comprise approximately 34 aa and mediate protein–protein interactions and the assembly of multiprotein complexes [41]. TvFKBP-63 contains an MukB domain (Figure 2H), which is found in MukB proteins and is associated with chromatin remodeling [42]. No other reports of FKBPs containing MukB domains were found.

Two of the three *T. vaginalis* Par proteins, namely TvPar17.8, and TvPar17.9, have similar molecular weights and 41.6% identity (Supplementary Table S1). Both proteins have a PpiC domain close to the C terminus, which covers most of the protein (~100 aa). In addition to this domain, the three TvPar proteins possess a WW domain of ~30 aa near the N terminus (Figure 2I,J).

The third TvPar, with a molecular weight of ~102 kDa (TvPar102), contains the PpiC domain, which spans ~100 aa near the N terminus. In addition, the WW domain near the N terminus contains a suppressor of forked (Suf) domain (Figure 2J), which is commonly associated with mRNA formation and polyadenylation in organisms such as *Drosophila melanogaster* [43]. Understanding the role of the Suf domain in TvPars could provide valuable insights.

TvPar102 is the largest Par identified among these clinically important parasites and is significantly larger than the typical Pars (~13 kDa). Its distinct C-terminal end, which consists of a series of α-helixes, sets it apart from other proteins in this group. However, its function remains unknown.

#### 2.1.2. *Entamoeba histolytica*

Amoebiasis is a parasitic disease caused by protozoan parasites belonging to the *Entamoeba* genus, among which *E. histolytica* is the most pathogenic species. This infection is transmitted through oral–fecal contamination, often via the consumption of food or water contaminated with parasite cysts, which migrate from the small intestine to the large intestine, where they divide by binary fission and are eliminated in the feces. Amoeboid forms may migrate out of the intestine and invade other body organs. This disease causes dysentery and various intestinal problems, affecting approximately 500 million people worldwide and causing more than one hundred thousand deaths per year [44].

The *E. histolytica* HM1-IMSS strain reference genome in the AmoebaDB database [25] (https://amoebadb.org/amoeba/app/, accessed on 1 June 2023) (Release 63 3 May 2023) contains six genes encoding CyPs, five genes encoding FKBPs, and two genes encoding Pars (Table 2).

Alignment of the EhCyP18 CLD with CLDs of CyPs from other organisms revealed high sequence conservation of this domain, in addition to the conservation of important aa residues in the active site (Supplementary Figure S3, [46]). Unlike the EhCyPs, the five FKBPs in *E. histolytica* are poorly conserved, which is unsurprising, since FKBPs are known to be highly divergent. The lowest sequence identities among the *E. histolytica* PPIases were found between its two Pars, EhPar13 and EhPar13.25, which share 20% identity, indicating significant divergence between these proteins.

The CyPs found in *E. histolytica* have molecular weights of between 10 and 40 kDa, and the CLD domain is consistently located at the N terminus. Interestingly, EhCyP10 is the smallest known CyP among parasites and contains a CLD. EhCyP21 and EhCyP22 contain an N-terminal sequence that allows them to localize to the ER. Notably, in addition to the CLD domain, CyPs EhCyP10 and EhCyP40 also contain an RRM domain of ~77–78 aa at the C terminus.

The five FKBPs of *E. histolytica* are globular proteins with molecular weights ranging from 18 to 46 kDa. Interestingly, in addition to the PPIase-FKBP domain, four of the FKBPs (EhFKBP-29, EhFKBP-35, EhFKBP-43, and EhFKBP-46) possess the TPR domain, which is related to protein–protein interactions. In contrast, EhFKBP-18 contains only the FKBP-like PPIase domain and a signal sequence at the N terminus with an unknown function. *E. histolytica* Pars contain only the PpiC-like PPIase domain; both Pars are small in size, which is common in the Pars identified to date. [47]

#### 2.1.3. *Giardia intestinalis*

Giardiasis, caused by protozoan parasite *Giardia intestinalis* (also known as *G. duodenalis* or *G. lamblia*), is a disease of global concern, affecting both developed and undeveloped nations. While asymptomatic cases are common, various intestinal and extraintestinal symptoms, as well as postinfection problems, have been recorded [48]. Giardiasis was designated as a neglected disease by the WHO in 2004, highlighting its public health significance [49]. It was estimated ~200 million cases of giardiasis worldwide in 2010 [50]. More recent reports in the European Union revealed that 18,000 cases were reported in 2019, with the highest incidence occurring in children between 0 and 4 years of age [51]. Likewise, in the United States alone, ~7 cases per 100,000 habitants were reported in 2019 [52].

*G. intestinalis* is a complex species comprising eight genetically related groups (assemblages A to H). Assemblages A and B, which are responsible for infecting humans, are subject to debate regarding their potential classification as different *Giardia* species due to significant genetic differences. Further research is required to resolve this question [53,54,55].

In this review, we focus on PPIases from the *G. intestinalis* genome reported in the GiardiaDB database (https://giardiadb.org/giardiadb/app, accessed on 1 June 2023) (Release 63 3 May 2023) [25], specifically from reference isolates WB (assemblage A), DH (sub-assemblage AII), and GS (assemblage B), which are all responsible for human infections [53,56,57]. These *G. intestinalis* isolates contain two CyPs (molecular weight < 25 kDa) and six FKBPs (molecular weight < 39 kDa), and no Pars were reported, similar in number to *Cryptosporidium* spp. and in contrast to parasites such as *T. vaginalis*, *Trypanosoma*, *Leishmania,* and *Toxoplasma,* which have more than 20 PPIases with molecular weights < 100 kDa.

**Figure 3:**
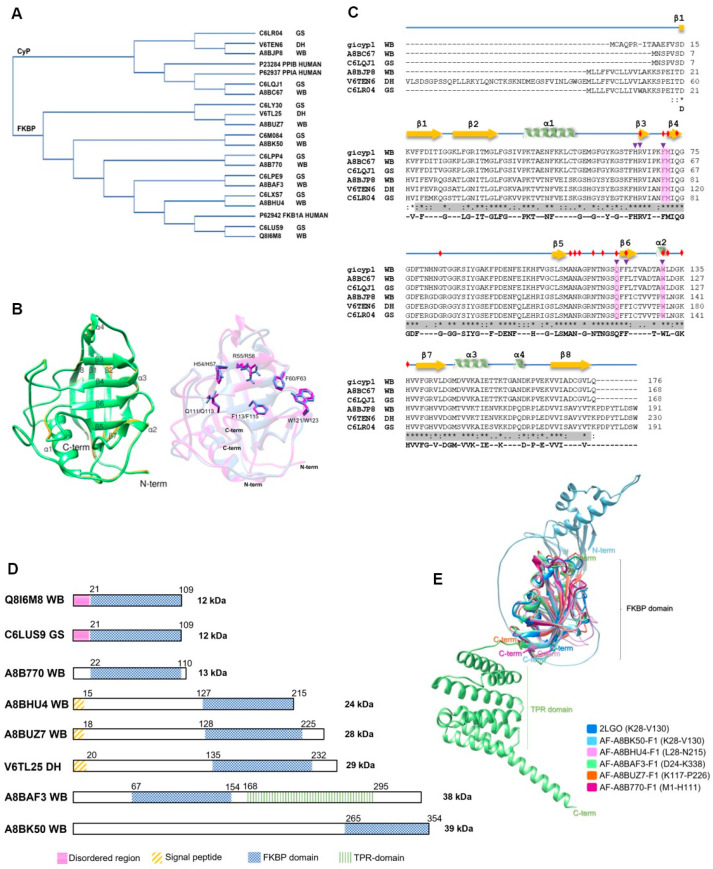
PPIases of the WB, GS, and DH *G. intestinalis* isolates. (**A**) Phylogram of PPIases from *G. intestinalis* and three human PPIases (PPIA/CyPA, PPIB/CyPB, and FKB1A/FKBP-12). (**B**) Overlapping structures of the GiCyP18 3D model of the WB (UniProt ID A8BC67 in green) and GS (UniProt ID C6LQJ1 in yellow) *G. intestinalis* isolates. Overlapping structures of hPPIA (1Ak4_A in magenta) and GiCyp18 (WB isolate in blue) are shown on the right. Active site residues taken from the Protein Domain Family of Conserved Domain Database (CDD) are represented by ball-and-stick illustration. (**C**) CyPs multiple sequence alignment. The secondary structure is depicted by yellow arrows for β-sheets and green ribbons for α-helixes. In gray is the cyclophilin-like domain (CLD). Red diamonds represent the putative CsA binding sites for GiCyP19. Purple triangles correspond to putative active sites. In pink are the binding pockets suggested for homology with hPPIA (UniProt ID P62937). * Represent conserved aa residues. (**D**) Domain architecture for FKBPs from *G. intestinalis*. The numbers above the bars indicate the amino acid position of each region. (**E**) Overlapping structures of FKBP domains from *G. intestinalis* WB isolate. The FKBP domains that were conserved in the alignment are indicated in parentheses. (**A**,**B**) were developed using the Clustal Omega 1.2.4, Clustal W and Clustal X tools [62,63]. The PDB and AlphaFold models were visualized with the UCSF Chimera 1.16 program [64]. All proteins have UniProt accession numbers (Supplementary Table S3, [34,62]).

The numbers of CyPs and FKBPs in the genomes of *Giardia* assemblages A and B are relatively consistent between the isolates. Some PPIases are the same in the two assemblages (Table 3), indicating potential similarities and a common ancestor (Figure 3A). In the DH isolate (sub-assemblage AII), the two GiCyPs and five GiFKBPs showed high sequence identity (>94.24%) compared to that of the WB isolate. However, differences in molecular weight were found for only one GiCyP and one GiFKBP (Table 3).

**Table 3:** Peptidyl-prolyl cis-trans isomerase repertoire from *Giardia intestinalis* ^1,2^.

Isolate	UniProt	GiardiaDB	NCBI	PDB	PPIase Name	Localization ^3^	Function ^3^	References
*G. intestinalis* WB					GiCyP19 (GiCyP1)			[58]
	A8BC67	GL50803_0017163	XP_001707838.1		GiCyP18	Cytoplasm Secreted	Virulence factor	[29,59,60]
	A8BJP8	GL50803_0017000	XP_001706629.1		GiCyP21	Cytoplasm Secreted		[29,59]
*G. intestinalis* DH	V6TEN6	DHA2_17000			GiCyP25	Membrane		[29]
*G. intestinalis* GS	C6LQJ1	GL50581_1019			GiCyP18	Secreted		[29]
	C6LR04	GL50581_1186			GiCyP21	Secreted		[43]
*G. intestinalis* WB	Q8I6M8	GL50803_10450	XP_001709141.1	2LGO	GiFKBP12	Secreted		[59,61]
	A8B770	GL50803_7246	XP_001709155.1		GiFKBP13	Cytoplasm		[29]
	A8BHU4	GL50803_101339	XP_001706925.1		GiFKBP24	Cytoplasm Secreted		[29,59]
	A8BUZ7	GL50803_42780	XP_001704692.1		GiFKBP28			
	A8BAF3	GL50803_3643	XP_001708385.1		GiFKBP38	Cytoplasm Secreted		[29,59]
	A8BK50	GL50803_10570	XP_001706462.1		GiFKBP39			
*G. intestinalis* DH	V6TL25	DHA2_151252			GiFKBP29			
*G. intestinalis* GS	C6LUS9	GL50581_2531			GiFKBP12	Secreted		[59]
	C6LPP4	GL50581_711			GiFKBP13			
	C6LXS7	GL50581_3593			GiFKBP24	Secreted		[59]
	C6LY30	GL50581_3701			GiFKBP28			
	C6LPE9	GL50581_614			GiFKBP38	Secreted		[59]
	C6M084	GL50581_4472			GiFKBP39			

^1^ *Giardia* assemblage A, isolate WB C6 (WB). *Giardia* sub-assemblage A2, isolate DH (DH). *Giardia* assemblage B, isolate GS/M, clone H7 (GS). ^2^ Previously reported names are presented in parentheses. ^3^ The localization and functions of PPIases were taken from the cited references or predicted by the Gene Ontology Consortium [29] in the UniProt database. Blank spaces: no reported data.

All *G. intestinalis* CyPs share a common CLD domain of ~89 aa, spanning 159 residues (Supplementary Table S2) (Figure 3C). The GiCyP18 proteins in the WB and GS isolates share 99.4% identity (Supplementary Table S3) and contain only a CLD domain (Supplementary Table S2) (Figure 3C). The GiCyP21 proteins in the WB and GS isolates share 94.2% identity (Supplementary Table S3) and have some characteristics in common with hCyPB [12]. These proteins contain an SP of 13 residues, 2 nonstructural regions at both ends of the CLD domain, and an additional 9 residues at the C terminus but lack the C-terminal ER sequence found in human hCyPB (Supplementary Table S2). In the DH isolate, GiCyP25 contains regions resembling those in hCyPB and a transmembrane helix (TMH) segment (Supplementary Table S2) (Figure 3C).

We also explored 3D GiCyP models using the AlphaFold database in UniProt (Release 2023_01). Despite the lack of crystallized GiCyPs to date, our analysis revealed that all the GiCyPs share a secondary and tertiary structure similar to that of hCyPA, as previously described [4,14]. Specifically, the secondary structure comprises eight β-strands, two α-helixes, two small helixes, and twelve regions of random-coil turns, which are also widely reported in other species (Figure 3C).

In the 3D structures, we observed that the central domain consists of eight β-strands flanked by an α-helix at each end of the barrel. These regions are connected by random-coil turn structures that are exposed on the protein surface. To determine the active site residues, we referenced the protein domain families in the Conserved Domains Database (CDD) [58] (Figure 3B). Notably, these active site residues coincide with those involved in CsA binding, including W123 in GiCyP1 (referred to as GiCyP19 in this review) (Table 3). GiCyP19 was the first CyP characterized from the *G. intestinalis* WB strain by Yu et al. in 2002 [65]. It should be noted that although the sequence of this gene had not yet been deposited in a database at the time of its publication, through BLAST analysis, we found an identity of around 98% of the sequence of the original publication with that of GiCyP19 found in the *Giardia* spp. genome.

*Giardia* FKBPs (Table 3) share similarities in terms of size and aa sequence among isolates (Supplementary Tables S2 and S3 [34,62]) (Figure 3D). The molecular weights of the proteins ranged from ~12 to 39 kDa, and their isoelectric points varied significantly from highly acidic at 4.6 to highly alkaline at 9.5 (Supplementary Table S2 [34]). Similar variations in size and charge have been observed in other organisms [13,66]. Among the *Giardia* FKBPs, only GiFKBP-12 has been crystallized (PDB: 2LGO) [61]. Its FKBP domain is located at the C-terminus and consists of 88 to 97 aa, forming six antiparallel β-strands and one α-helix. It contains conserved residues responsible for inhibitor binding (Figure 3D) [67]. Other Giardia FKBPs possess additional regions along with the FKBP domain (Table 3). Some have a small, disordered region of approximately 21 aa at the N terminus (GiFKBP-12), while others have an SP of 15 or 18 aa (GiFKBP-24, GiFKBP-28, and GiFKBP-29). Only one FKBP (GiFKBP-38) contains a TPR region (Supplementary Table S2) formed by six antiparallel α-helixes (Figure 3E).

### 2.2. Trypanosomatid Parasites

#### 2.2.1. *Trypanosoma cruzi* and *Trypanosoma brucei gambiense*

*T. cruzi* and *T. brucei gambiense* are protozoan parasites that cause significant health impacts through trypanosomiasis. *T. cruzi* causes Chagas disease (also known as American trypanosomiasis), which is transmitted to humans and other mammals by Triatominae insects. *T. brucei gambiense* and *T. brucei rhodesiense* cause sleeping sickness (also known as African trypanosomiasis), which is transmitted by the tsetse fly [68]. According to the WHO, between 6 and 7 million people worldwide, the majority in Latin America, are infected by *T. cruzi,* including around 30,000 and 40,000 new infections and between 10,000 and 20,000 deaths every year. At the same time, it is estimated that ~75 million people are at risk of infection [69]. For example, in 2016 to 2018 in Brazil the average chronic disease occurrence rate is 3.2 per 100,000 people per year. Furthermore, a total of 350 deaths are recorded annually (male predominance, age ≥60 years, and chronic disease with cardiac involvement as the main mortality cause), with an annual average mortality proportion of 1.6 per 100,000 people [70]. Moreover, African trypanosomiasis is endemic in numerous African regions, putting 70 million people at risk throughout sub-Saharan Africa. *T. brucei gambiense* accounts for more than 95% of the reported cases of African trypanosomiasis [69]. This infection is mainly present in Western and Central Africa. It has a chronic progressive course lasting over three years, mimicking hematological conditions [71].

The CyPs in the *T. cruzi* CL Brener Esmeraldo-like genome have been reported in the TriTrypDB database (https://tritrypdb.org/tritrypdb/app/, accessed on 1 April 2023) (Release 62 9 Mar 2023) [25] and include 15 paralogs [72]. We analyzed the 3D structure of these CyPs using the predicted structures generated by AlphaFold in the UniProt database (Release 2023_01). All these CyPs share the highly conserved CLD domain, an eight-stranded, antiparallel ꞵ-barrel structure accompanied by two α-helixes, and show some differences in regions outside the CLD domain. TcCyP19 consists predominantly of the CLD domain, whereas the other fourteen are isoforms (Table 4). For instance, TcCyP21 and TcCyP24 have SPs of 26 and 25 aa residues in length, respectively. Notably, TcCyP21 is the only *T. cruzi* CyP with a reported crystal structure (PDB:1XO7) [61]. Its PDB ID is linked to a different UniProt ID (Q4DPB9) due to its origin in the *T. cruzi* CL Brener non-Esmeraldo-like genome. However, the alignment of the two protein sequences revealed 97.9% identity.

Furthermore, TcCyP22, TcCyP26, TcCyP30, and TcCyP42 contain an elongated region in the N terminus, while TcCyP20 has a small, elongated region in the C terminus. Additionally, TcCyP25, TcCyP28, TcCyP29, TcCyP35.3, and TcCyP35 (previously named TcCyP34 by Potenza et al., 2006) [73] exhibit elongated regions in both the N- and C-terminal segments. Moreover, TcCyP38 (previously named TcCyP40) [73] contains the TPR motif. Finally, TcCyP103 (previously named TcCyP110) [73] is the largest CyP (103 kDa) and contains an array of structures adjacent to the CLD domain, which were identified as disordered regions with both basic and acidic residues.

**Table 4:** Peptidyl-prolyl cis-trans isomerase repertoire from *Trypanosoma cruzi* ^1^.

Parasite	UniProt	TriTrypDB	NCBI	PDB	PPIase Name	Localization ^2^	Function ^2^	References
*T. cruzi* CL Brener	Q4E4L9	TcCLB.506925.300 (CYPA)	XP_821578.1		TcCyP19	Extracellular space	Promotes ROS production in host cells	[72,74,75,76,77]
	Q4DC03	TcCLB.507009.100	XP_811912.1		TcCyP20			
	Q4DNC9	TcCLB.507521.70	XP_815879.1	1XO7	TcCyP21			[72,76]
	Q4DI85	TcCLB.504035.70	XP_814080.1		TcCyP22	Mitochondria	Cell death regulation	[72,78]
	Q4CXV1	TcCLB.506413.80	XP_806960.1		TcCyP24			
	Q4DFL3	TcCLB.508323.94	XP_813175.1		TcCyP25			[72,76]
	Q4D4K3	TcCLB.503885.40	XP_809302.1		TcCyP26			
	Q4CX88	TcCLB.509499.10	XP_806737.1		TcCyP28			[72,76]
	Q4DQI8	TcCLB.505807.10	XP_816616.1		TcCyP29			
	Q4DNS3	TcCLB.511589.50	XP_816007.1		TcCyP30	Membrane		[29,72]
	Q4DM35	TcCLB.511577.40 (CYP35)	XP_815421.1		TcCyP35 (TcCyP34)			[72,76]
	Q4DVC9	TcCLB.511217.120	XP_818332.1		TcCyP35.3 (TcCyP35)			[72]
	Q4E4G0	TcCLB.506885.400 (CYP40)	XP_821542.1		TcCyP38 (TcCyP40)			[29,72]
	Q4DG41	TcCLB.510761.44	XP_813344.1		TcCyP42	Membrane		[29,72]
	Q4D1M5	TcCLB.504215.10	XP_808273.1		TcCyP103 TcCyP110			[72]
*T. cruzi* Y	Q09734	TcYC6_0113560	CAA49346.1	1JVW	TcFKBP22 (TcMIP)	Extracellular space	Host cell entry/invasion	[73,79]
*T. cruzi* CL Brener	Q4D5W5	TcCLB.508169.69	XP_809772.1		TcFKBP12			
	Q4DFL5	TcCLB.508323.84	XP_813174.1		TcFKBP12.2			
	Q4D7F5	TcCLB.511731.89	XP_810317.1		TcFKBP35			
	Q4CZN2	TcCLB.511353.10	XP_807578.1		TcFKBP52			
	Q4CYE6	TcCLB.507629.39	XP_807152.1		TcFKBP93			
*T. cruzi* CL Brener	Q4D8F7	TcCLB.508567.70 (Pin1)	XP_810661.1		TcPar12.6 (TcPin1)	Cytosol		[80,81]
	Q4D394	TcCLB.506697.50	XP_808848.1		TcPar13 (TcPar14)			[82]
	Q4D9J4	TcCLB.506857.60 (Par45)	XP_811046.1		TcPar45	Nucleus		[82]

^1^ Previously reported PPIasa names are presented in parentheses. ^2^ The localization and functions of PPIases were taken from the cited references or from the UniProt database and were predicted by the Gene Ontology Consortium [29]. Blank spaces: data not reported.

The TriTrypDB database includes six *T. cruzi* FKBP genes [72] (Table 4). Structural analysis revealed that these FKBPs range in size from 12 to 93 kDa and share similarities in the catalytic domain. Two FKBPs, namely TcFKBP-12 and TcFKBP-12.2, consist mostly of the FKBP domain. The four remaining proteins contain an FKBP domain plus other motifs. TcFKBP-22 (also referred to as TcMIP, the Microphage Infectivity Potentiator, by Moro et al., 1995) [79] contains a 29 aa SP. TcFKBP-22 is from *T. cruzi* strain Y and is the only *T. cruzi* FKBP that has been characterized and crystallized (PDB: 1JVW) [61]. TcFKBP-35 has extensive elongation in its C terminus, and TcFKBP-52 and TcFKBP-93 have elongated regions in their N termini that have been identified as coiled-coils. These coiled-coils are involved in various biological functions as molecular spacers within proteins, influencing the architecture of organelles such as centrioles and the Golgi apparatus and facilitating the binding of transport vesicles to the Golgi apparatus [80].

Finally, *T. cruzi* contains three Pars [72] (Table 4) ranging from 12 to 45 kDa, all possessing the PpiC domain. TcPar12.6 (previously named TcPin1 by Erben et al., 2007 [82] and a homolog of hPin1) consists entirely of the PpiC domain and lacks the protein–protein interaction-related WW domain at the N terminus. In addition, TcPar13 (previously named TcPin14 by Erben et al., 2010 [83] and a homolog of hPar14) has an elongated region in its N terminus, which is described as disordered and lacks the WW domain. Unlike TcPar13, hParv14 lacks the N-terminal WW domain but has an unstructured N-terminal extension that is essential for its nuclear localization and DNA binding. The third Par, TcPar45, has an elongated N-terminal segment and contains a fork-head-associated domain (FHA) instead of a WW domain; FHA domains play a role in recognizing phosphopeptides related to biological processes [84].

The genome of *T. brucei gambiense* strain DAL972 is closely related to the *T. brucei* genome, which suggests that the DAL972 genome is an effective scaffold for any *T. brucei* genome sequence [74]. The genome of *T. brucei gambiense* strain DAL972 in TriTrypDB (Release 62 9 Mar 2023) [25] encodes 19 members of the CyP family (four more than *T. cruzi*), 6 members of the FKBP family, and 3 members of the Pars family [75] (Table 5).

We analyzed the 3D models of these PPIases and observed similarities with those in *T. cruzi*. The TbgCyPs exhibit elongated N- or C-terminal segments or both (TbgCyP21.1, TbgCyP21.4, TbgCyP25.55, TbgCyP27.1, TbgCyP27.4, TbgCyP29, TbgCyP30, TbgCyP33, TbgCyP43, TbgCyP46, and TbgCyP100). TbgCyP38, like TcCyP38, contains a TPR motif. Furthermore, four CyPs have SP domains (TbgCyP21.2, TbgCyP24, TbgCyP25.56, and TbgCyP58). *T. cruzi* does not contain a 58 kDa CyP, unlike *T. brucei gambiense*. TbgCyP58 also contains an RRM motif involved in nucleic acid and/or protein recognition. *T. brucei gambiense* has three CyPs (TbgCyP19, TbgCyP20.3, and TbgCyP20.5) that consist predominantly of the CLD domain, whereas *T. cruzi* has only one CyP with this domain.

We found that the *T. brucei gambiense* FKBPs also contain elongated N- and C-terminal regions (TbgFKBP12.3 and TbgFKBP36), disordered regions and a coiled coil (TbgFKBP92), and an SP (TbgFKBP21). Additionally, TbgFKBP48 contains a disordered region paired with coiled-coils and a TPR motif. Only TbgFKBP12 consists almost exclusively of the FKBP domain.

The *T. brucei gambiense* Pars are also homologous to *T. cruzi* Pars. TbgPar12 and TbgPar13 share more than 68% sequence identity with TcPar12.6 and TcPar13, respectively. TbgPar12 consists mainly of the PpiC domain, while TbgPar13 contains an elongated N-terminal segment that is reported to be disordered. Moreover, TbgPar42 shares 60% sequence identity with TcPar45 and contains both a PpiC domain and an FHA domain.

#### 2.2.2. *Leishmania major* and *Leishmania donovani*

Leishmaniasis is caused by protozoan parasites in the *Leishmania* genus, which are transmitted to humans and other animals via the bites of infected sandfly mosquitos. Leishmaniasis is present in 88 countries around the world, most of them being developing nations, with a prevalence of at least 12 million infected people and a population at risk of almost 350 million people [89]. It is also estimated that there is an annual incidence of 600,000 to 1 million cases of cutaneous leishmaniasis and ~50,000–90,000 cases of visceral leishmaniasis [90]. *L. major* and *L. donovani* are linked to cutaneous and visceral leishmaniasis, respectively, in Asia, Africa, and parts of Europe [91].

The TriTrypDB database (https://tritrypdb.org/tritrypdb/app/, accessed on 1 June 2023) (Release 63 3 May 2023) [25] contains at least 24 PPIase genes from the *L. major* isolate Friendlin reference genome [92] and the same number from the *L. donovani* BPK282A1 reference genome [93]. Both species contain 17 CyPs, 5 FKBPs, and 2 Pars (Table 6 and Table 7). Despite their high overall sequence identity and similar molecular weights, LmPar47 and LdPar17 exhibit only 34% sequence identity, mainly due to differences in molecular weight (Supplementary Table S4). Interestingly, the gene encoding LdCyP108, categorized as a conserved hypothetical protein in the *Leishmania* database (TriTrypDB), was confirmed to be a CyP through further verification via the UniProt and NCBI databases. The number of PPIases in *Leishmania* is comparable to that in other trypanosomatids, such as *T. cruzi* and *T. brucei* (Table 4 and Table 5).

The first PPIases discovered in *L. major* and *L. donovani* were CyPs identified during studies involving Cyclosporin A (CsA) [96,99]. *Leishmania* CyPs vary in size from 19 to 108 kDa and have additional N-terminal or C-terminal extensions or both alongside the CLD domain [90]. Three *Leishmania* CyPs stand out in particular, namely LmCyP24.6 (also known as LmaCyP5), which features a PLD (prokaryotic lipid attachment domain); LmCyP38 (also known as LmaCyP40), which is distinguished by two additional TPR domains; and LdCyP38.4 (also known as LdCyP40), which contains a TPR domain at its C terminus.

The crystal structure of LdCyP20 (PDB: 2HAQ) from *L. donovani* closely resembles that of other CyPs, with an eight-stranded β-barrel and two α-helixes, albeit with minor differences from hCyPA [97]. LmCyP32 from *L. major* (PDB: 2HQJ) shares the same secondary structure, with slight variations in the loops. Additionally, *L. major* has two more crystallized CyPs, namely LmCyP25 (PDB: 7AIH) and LmCyP29 (PDB: 7AM2), both of which are part of a large subunit of the *L. major* mitoribosome [94].

The FKBPs identified in both *L. major* and *L. donovani* display notable similarities. These proteins share the FKBP PPIase domain and fall within the 11.8 to 48 kDa range. Interestingly, LmFKBP48 and LdFKBP47 contain an additional TPR domain at the C-terminus. Among *Leishmania* Pars, LmPar13, LdPar12, and LdPar17 are characterized by a PpiCPPIase domain and similar molecular weights. In the case of LmPar47, an FHA domain is present alongside the PpiC domain.

### 2.3. Apicomplexan Parasites

#### 2.3.1. *Plasmodium falciparum* and *Plasmodium vivax*

The *Plasmodium* genus causes malaria, which is transmitted by infected female *Anopheles* mosquitoes. *P. falciparum* and *P. vivax* are the most prominent species that cause malaria in humans due to their characteristics and impact on public health. *P. falciparum* is the most prevalent pathogen in Africa, and *P. vivax* is the dominant parasite in most countries outside of sub-Saharan Africa. According to the latest report, there were 247 million cases of malaria in 2022 and 608,000 malaria deaths in 85 countries in 2022, with 80% of deaths occurring in children under 5 years of age [104].

The reference genome of the *P. falciparum* 3D7 isolate is widely used in malaria research. According to the PlasmoDB database (https://plasmodb.org/plasmo/app, accessed on 1 June 2023) (Release 63 3 May 2023) [25], this genome contains the 13 genes encoding PPIases, namely 11 CyPs and 2 FKBPs. To date, no Pars have been reported (Table 8). The new reference genome for *P. vivax* was obtained from the P01 isolate [105]. According to PlasmoDB (Release 63 03 May 2023), this genome contains the same number of PPIases as *P. falciparum*. Thus, this family is conserved between species even though the *P. vivax* genome presents almost twice the genetic diversity of *P. falciparum* [106,107].

**Figure 4:**
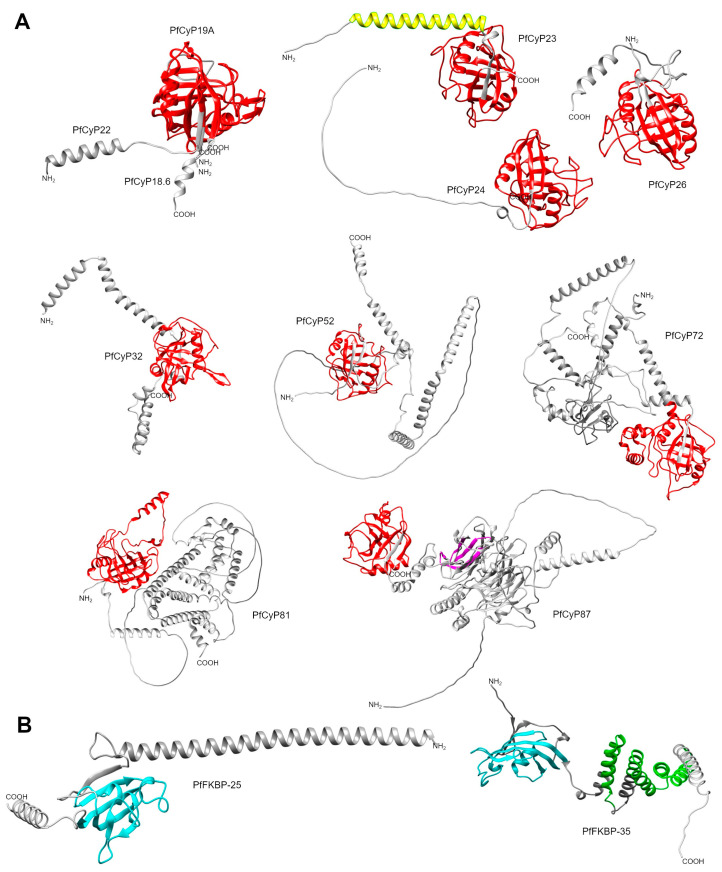
The 3D structures of *P. falciparum* PPIases. (**A**) Cyclophilins. The CLD domain is shown in red, the coiled coil in yellow, and the WD40 region in magenta. (**B**) FKBPs. The FKBP domain is shown in cyan, and the TPR region is shown in green. *P. vivax* 3D models are very similar to *P. falciparum* PPIases. The PDB 1QNG (PfCyP19A) and AlphaFold models were visualized using the UCSF Chimera 1.16 program [64].

The *P. falciparum* CyPs have molecular weights ranging from 19 to 87 kDa. They contain a conserved CLD domain with a typical eight-strand β-barrel and two α-helixes. Most of the PfCyPs contain a CLD region of ~142–165 aa, but the largest CLD region is 209 aa (PfCyP81) (Table 8). PfCyP19 and PfCyP18.6 (also known as PfCyP19C) consist only of the CLD domain, and the rest are CyP isoforms. For example, PfCyP22 contains an SP, and PfCyP23 has a coiled coil near the N terminus. PfCyP25 has N-terminal extensions, whereas PfCyP26 and PfCyP53 have C-terminal extensions, and PfCyP32 and PfCyP72 have extensions in both the N- and C-terminal regions. PfCyP87 contains a WD40 repeat, and PfCyP81 contains a region related to the SYF2 family (Figure 4A,B). This analysis is similar to that reported by Marín-Menéndez and Bell (2011) [108], with some differences in the length of regions or domains. Additionally, two crystallized structures of the CyP catalytic domain have been reported for *P. falciparum*, namely PfCyP19 (PDB: 1QNG) and PfCyP87 (PDB: 2FU0) [109] (Table 8).

**Table 8:** Peptidyl-prolyl cis-trans isomerase repertoire from *Plasmodium falciparum* ^1,2^.

UniProt	PlasmoDB	NCBI	PDB	PPIase Name	Localization ^3^	References
Q8IIK3	PF3D7_1116300 (CYP19C)	XP_001347841.1		PfCyP18.6 (PfCyP19C)	Nucleus (Spliceosome)	[29,107,110]
Q76NN7	PF3D7_0322000 (CYP19A)	XP_001351290.1	1QNG	PfCyP19 (PfCyP19A)	Cytoplasm	[29,107,110,111,112,113]
Q8IIK8	PF3D7_1115600 (CYP19B)	XP_001347835.1		PfCyP22 (PfCyP19B)	Cytoplasm Membrane	[29,107,113,114,115]
Q8I3I0	PF3D7_0528700 (CYP23)	XP_001351841.1		PfCyP23	Nucleus (Spliceosome)	[29,102,107,109,110]
Q8I6S4	PF3D7_0804800 (CYP24)	XP_001349469.1		PfCyP25(PfCyP24)	Membrane	[29,107,110,116,117]
Q8I621	PF3D7_1202400 (CYP26)	XP_001350433.1		PfCyP263	Cytoplasm	
Q8I5Q4	PF3D7_1215200 (CYP32)	XP_001350556.1		PfCyP32	Cytoplasm and Mitochondria	[29,107,110]
Q8ILM0	PF3D7_1423200 (CYP52)	XP_001348397.2		PfCyP53 (PfCyP52)	Nucleus (Spliceosome)	[29,107,110]
Q8I2K8	PF3D7_0930600 (CYP72)	XP_001352173.1		PfCyP72	Nucleus	[11,29]
Q8IAN0	PF3D7_0803000 (CYP81)	XP_001349484.1		PfCyP81	Nucleus	[11,29]
Q8I402	PF3D7_0510200 (CYP87)	XP_001351660.1	2FU0	PfCyP87	Nucleus (Spliceosome)	[29,107,108]
C0H5B2	PF3D7_1313300	XP_002809009.1		PfFKBP25.6		
Q8I4V8	PF3D7_1247400	XP_001350859.1	2OFN	PfFKBP35	Cytoplasm and Nucleus	[29,109,118,119,120]

^1^ Isolate: *Plasmodium falciparum* 3D7. ^2^ Previously reported PPIAse names are presented in parentheses. ^3^ The localization and functions of PPIases were taken from the cited references or predicted by the Gene Ontology Consortium [29] in the UniProt database. ER: endoplasmic reticulum. Blank spaces: no reported data.

To date, no *P. vivax* CyPs have been characterized (Table 9). Approximately half the CyP sequences of the two *Plasmodium* species exhibited ≥80% identity. However, PvCyP29, PvCyP52, PvCyP65, PvCyP71, and PvCyP87 have below-average identity (Supplementary Table S5). PvCyP19 consists predominantly of the CLD domain. Outside this domain, the *P. vivax* homolog CyPs show similarities to PfCyPs. For example, PvCyP21 contains an SP, and PvCyP83 contains a WD40 repeat. The CyPs with elongated segments in the N terminus, C terminus, or both are PvCyP18, PvCyP23, PvCyP26, PvCyP29, PvCyP32, and PvCyP52. Intriguingly, *P. vivax* has two CyPs with distinct molecular weights, namely PvCyP29 and PvCyP65, instead of PfCyP25 and PfCyP81. PvCyP65 also contains a region belonging to the SYF2 family, as suggested for PfCyP81.

*P. falciparum* and *P. vivax* possess only two FKBPs, in contrast to most of the other mentioned parasites, which typically have at least five FKBPs. Some of the *Plasmodium* FKBPs have 60% identity (PfFKBP25.6 and PvFKBP25) and 80% identity (PfFKBP35 and PvFKBP34) (Supplementary Table S5). The conserved FK506 binding domains of three *Plasmodium* FKBPs have been crystallized, namely PfFKBP35 (PDB: 2OFN) [116,121], PvFKBP25 (PDB: 4JYS) [122], and PvFKBP34 (PDB: 2KI3) [123] (Table 8 and Table 9). This domain shares many of its secondary structures, comprising a six-stranded β-sheet and short α-helixes, with an additional β-strand at the N terminus. In addition to this domain, PfFKBP25 and PvFKBP25 contain extensions at the N terminus. PvFKBP25 is considered an atypical FKBP that lacks catalytic activity and does not have the conserved active site in typical FKBPs [118,122]. PfFKBP35 and PvFKBP34 have three and one TPR domains, respectively. These domains control the dimeric form of PfFKBP35, while the FKBP domain remains a monomer in solution [111].

**Table 9:** Peptidyl-prolyl cis-trans isomerase repertoire from *Plasmodium vivax* ^1,2^.

UniProt	PlasmoDB	NCBI	PDB	PPIase Name	Localization ^3^	References
A0A1G4HCW7	PVP01_0916900 (CYP19C)	XP_001615280.1		PvCyP18.5		
A0A1G4HBM6	PVP01_0818200 (CYP19A)	XP_001614493.1		PvCyP19		
A0A1G4HCM3	PVP01_0916400	XP_001615276.1		PvCyP21		
A0A1G4HDR7	PVP01_1005100 (CYP23)	XP_001613671.1		PvCyP23		
A0A1G4H2Q1	PVP01_1301700 (CYP26)	XP_001616500.1		PvCyP26		
A0A1G4GR33	PVP01_0115700 (CYP24)	XP_001608574.1		PvCyP29		
A0A1G4H4X8	PVP01_1434000 (CYP32)	XP_001617250.1		PvCyP32		
A0A1G4HIV6	PVP01_1325800	CAG9475874.1		PvCyP52		
A0A1G4HAY2	PVP01_0729200	XP_001614845.1		PvCyP71		
A0A1G4GR20	PVP01_0117200 (CYP81)	CAG9485095.1		PvCyP65	Nucleus	[29]
A0A1G4HEA6	PVP01_1023800 (CYP87)	XP_001613274.1		PvCyP83		
A0A1G4H4D0	PVP01_1414200	XP_001617060.1	4JYS	PvFKBP25 ^3^		[116]
A0A565A3M9	PVP01_1464500	XP_001613999.1	2KI3	PvFKBP34 ^3^ (PvFKBP35)		[120,122]

^1^ Isolate: *Plasmodium vivax* P01. ^2^ Previously reported PPIAse names are presented in parentheses. ^3^ The localization and functions of PPIases were taken the cited references or predicted by the Gene Ontology Consortium [29] in the UniProt database. Blank spaces: no reported data.

#### 2.3.2. *Toxoplasma gondii*

Toxoplasmosis is caused by the parasite *Toxoplasma gondii* in warm-blooded animals, including humans. Most infected individuals with strong immune systems do not show symptoms and do not require treatment. However, pregnant women and immunocompromised individuals need to be cautious, as toxoplasmosis can cause severe health problems [124]. According to the latest U.S. CDC report, more than 40 million people in the United States are infected by this parasite [125]. The worldwide incidence of congenital infection is estimated to be 1.5 cases/1000 live births, with a higher burden in South America and some Middle Eastern and low-income countries and a lower burden in European countries [126]. In 2021, the ECDC reported that the incidence of congenital toxoplasmosis in the European Union/EEA was 5.51 cases per 100,000 live births [127].

The *T. gondii* ME49 isolate is type II and considered the priority type due to having the closest association with human disease [128]. According to the ToxoDB database (https://toxodb.org/toxo/app, accessed on 1 June 2023) (Release 63 3 May 2023) [25], the *T. gondii* ME49 genome contains 20 genes encoding PPIases, 13 of which are CyPs, 4 of which are FKBPs, 2 of which are Pars, and 1 of which is a dual PPIase (FKBP-CyP) (Table 10).

The size of CyPs in *T. gondii* ranges from 18 to 86 kDa. Two crystallized CyP proteins, namely TgCyP64 (PDB: 3BKP) [61] and TgCyP69 (PDB: 3BO7) [61], exhibit conserved secondary structures within the CLD domain, featuring eight β-strands and two α-helixes. TgCyP18 mainly consists of the CLD domain, and the rest of the CyPs are isoforms. TgCyP20 contains an SP, and TgCyP21, TgCyP23, TgCyP26, TgCyP32, TgCyP35, and TgCyP38 have a TMH domain and extra extensions at the N or C terminus or both, in addition to the CLD domain. TgCyP38 initially appeared to be CyP20 due to its molecular weight, as previously reported [129]. However, a closer analysis revealed that the ~20 kDa region corresponds to the CLD. This finding suggests that this protein undergoes a specific post-translational modification process distinct from glycosylation to remove its N-terminal extension. TgCyP38 also has a transmembrane domain that appears to play an important role in its folding, assembly, and function [128].

**Table 10:** Peptidyl-prolyl cis-trans isomerase repertoire from *Toxoplasma gondii* ^1^.

UniProt	ToxoDB	NCBI	PDB	PPIase Name	Localization ^3^	Function ^3^	References
A0A125YZ79	TGME49_289250	XP_018636397.1		TgCyP18		Manipulates host cell responses	[130,131,132]
S8F7V1	TGME49_221210	XP_002369951.1		TgCyP20	Secreted	Manipulates host cell responses	[129,133]
A0A125YV51	TGME49_270560	XP_002365722.1		TgCyP21			
A0A125YL73	TGME49_285760	XP_002369214.1		TgCyP23			[132]
A0A125YLU4	TGME49_230520	XP_002367963.2		TgCyP26			
S8FB56	TGME49_238000	XP_018637703.1		TgCyP32			
A0A125YQ35	TGME49_262520	XP_002365354.1		TgCyP35			
S8F5I7	TGME49_205700	XP_002367801.1		TgCyP38	Membrane		[29,129]
A0A125YVH7	TGME49_241830	XP_002366733.1	3BKP	TgCyP64			[61]
A0A125YII8	TGME49_229940	XP_002367918.1		TgCyP66.21	Nucleus		
S8GFQ1	TGME49_227850	XP_002366408.1		TgCyP66.25	Nucleus		
A0A125YUW2	TGME49_305940	XP_002370366.1	3BO7	TgCyP69			[61]
S8FD30	TGME49_320640	XP_002369921.1		TgCyP86			
S8GFX3	TGME49_228360 FKBP-12	XP_002366458.1		TgFKBP38	Membrane		[29]
S8F5H8	TGME49_285850	XP_002369223.1		TgFKBP46			
Q4VKI5	TGME49_283850	XP_018637740.1		TgFCBP57 ^2^			[134]
A0A125YIR1	TGME49_318275	XP_018637996.1		TgFKBP64			
S8F128	TGME49_258625	XP_018637023.1		TgFKBP66			
A0A125YRG0	TGME49_258930	XP_002365107.1		TgPar13			
S8EUZ2	TGME49_228040	XP_002366427.1		TgPar96			

^1^ Isolate: *Toxoplasma gondii* ME49. ^2^ TgFCBP57 exhibited two activities: an FK506 and cyclosporin-binding protein. ^3^ The localization and functions of PPIases were taken from the cited references or predicted by the Gene Ontology Consortium [29] in the UniProt database. Blank spaces: no reported data.

Moreover, TgCyP66.2 has a coiled-coil structure associated with the 2YF2 family due to sequence similarities, while TgCyP66.25 contains an RRM. Additionally, TgCyP69 contains a U-box domain. TgCyP86, the largest CyP in this organism (Table 10), contains a WD40 repeat, similar to the largest CyPs in other parasites, such as *P. falciparum* and *P. vivax.*

**Figure 5:**
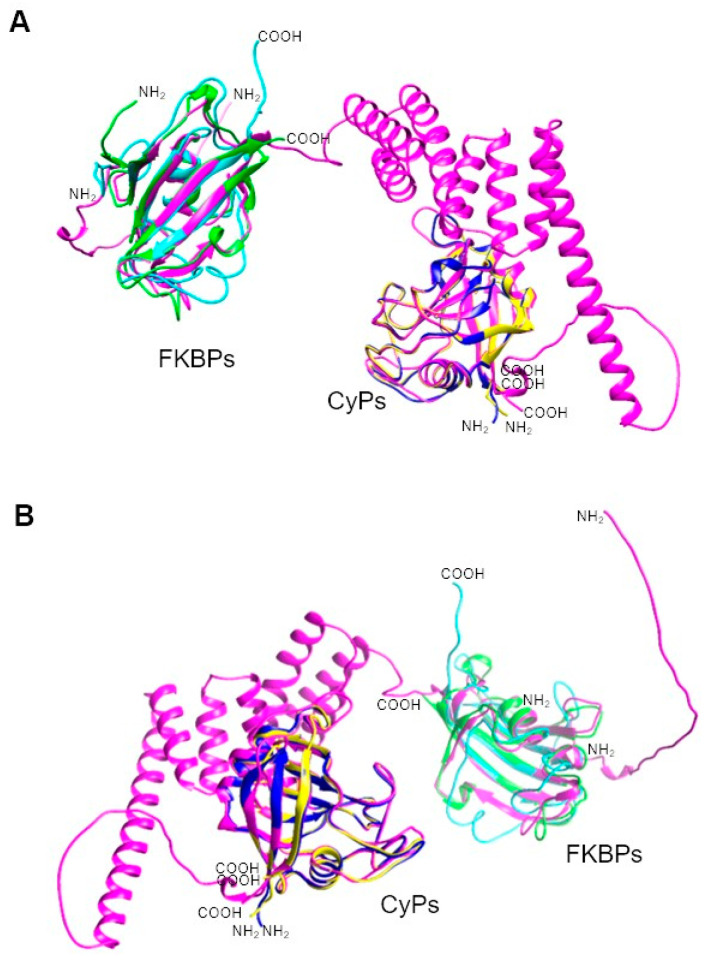
Comparison of the 3D structures of *P. falciparum* and *P. vivax* PPIases FKBP-35 and CyP19A with the *Toxoplasma gondii* dual PPIase (TgFCBP-57). (**A**) Front view. (**B**) Back view. The 3D structure of TgFCBP57 is shown in violet, and the 3D structures of PfFKBP-35 (PDB: 2OFN) and PvFKBP-35 (PDB: 2KI3) are shown in cyan and green, respectively. The 3D structures of PfCyP19 (PDB: 1QNG) and PvCyP19 are shown in yellow and blue, respectively. The PDB and AlphaFold models were visualized with the UCSF Chimera 1.16 program [64].

*T. gondii* FKBPs range in size from 38 to 67 kDa and share similarities at the structural level, primarily due to their FKBP domain. Notably, none of these FKBPs has TPRs, unlike those in other parasites. An intriguing PPIase in *T. gondii* is TgFCBP57, which is classified as a dual-family PPIase because it possesses both FKBP and CyP domains at the N and C terminus, respectively, linked by TPRs [134] (Table 10). Figure 5 shows that the PPIase domains of other Apicomplexa parasites, such as *P. falciparum* and *P. vivax*, are highly conserved, even for this dual PPIase, showing very similar three-dimensional structures. Although an RNAi study showed that PPIases are essential for *T. gondii* growth [134], the specific function(s) of the FKBPs has/have yet to be determined.

Two members of the Parv family are present, namely TgPar13 and TgPar96 (Table 10). TgPar13 comprises an entire PpiC domain. TgPar96 is the second largest Parv after TvPar102. This Par is 912 aa long and contains a PpiC domain within the last 146 aa, along with an FHA domain. However, the sequences outside the catalytic domain have not been well characterized. These structural and sequence resemblances among parasite PPIases demonstrate their shared evolutionary history.

#### 2.3.3. *Cryptosporidium parvum* and *Cryptosporidium hominis*

*Cryptosporidium spp*. is the causal agent of the infection known as cryptosporidiosis, a diarrheal-type infection that can include symptoms such as vomiting or nausea, with self-limiting symptoms in immunocompetent patients and more severe symptoms in immunocompromised patients. The main Cryptosporidium species known to affect humans are *C. parvum* and *C. hominis*, which cause more than 90% of infections [135].

Cryptosporidiosis is considered one of the most lethal diseases for infants under two years of age, who are usually also affected by malnutrition [136]. In Africa and Asia alone, there are ~7.5 million cryptosporidiosis cases each year, with more than 200,000 deaths caused by *Cryptosporidium* spp. [137].

Analysis of the Cryptosporidium genome (https://cryptodb.org/cryptodb/app, accessed on 9 July 2024), particularly *C. parvum* (Release 52, 20 May 2021) and *C. hominis* (Release 52, 20 May 2021), showed a total of nine PPIases in both cases. Seven are cyclophilin-type PPIases, and two are FKBP-type PPIases, with no parvulins found. The number of PPIases is much lower than that found in the other Apicomplexa species analyzed in this work. However, the range of their molecular weights is consistent with those from other parasite PPIases, ranging between 18 and 89 kDa (Table 11 and Table 12).

**Figure 6:**
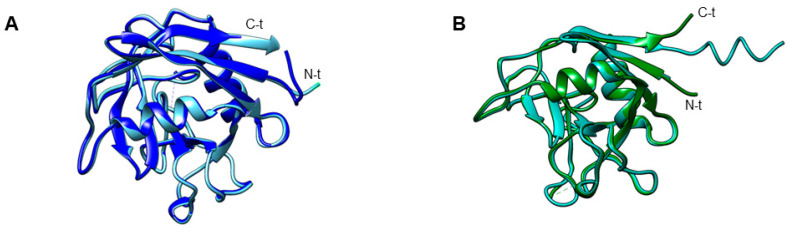
Structural alignment between cyclophilins of *C. parvum* and *C. hominis*. (**A**) Comparison of the crystal structure of CpCyP18 (PDB: 2PLU, blue) and the predicted structure of ChCyP18 (cyan). (**B**) Comparison of the crystal structure of CpCyP19 (PDB: 2POE, green) and the predicted structure of ChCyP19 (light green). The PDB and AlphaFold models were visualized with the UCSF Chimera 1.16 program [64].

Furthermore, it was observed that the number and size of PPIases were very similar between the two *Cryptosporidium* spp. Therefore, sequence analyses were carried out by pairwise alignment. It was found that these PPIases shared very high sequence identities (over 88%; Supplementary Table S6). This homology in the protein sequences indicates that these proteins could come from orthologous genes. This is also corroborated by the structural comparison analyses, which showed 97% identity between the crystal structure of CpCyP18 (PDB:2PLU) [61] and the structure predicted by AlphaFold [139] for ChCyP18, with minor differences observed in the lengths of the N and C termini. In both proteins, seven β-strands and three α-helixes were observed (Figure 6A). The same type of structures were also observed between the 19 kDa CyPs of both parasites, with an identity of ~90% in structural alignment and the greatest difference observed in the length of the C-terminal end (Figure 6B). These structural and sequence similarities between the PPIases of the two *Cryptosporidium* species could indicate that they fulfill the same functions in both parasites.

## 3. Localization and Functions of PPIases in Parasites

The localization and functions of PPIases in clinical protozoan parasites are not yet well understood. In this section, we provide a broad outline of the importance of these PPIase isoform-specific extensions, which can provide valuable insights into the precise mechanisms by which PPIases regulate essential cellular processes, making them attractive targets for further research and potential therapeutic interventions.

*T. vaginalis* contains only two characterized CyPs, namely TvCyP19 and TvCyP19.9 (known as TvCyP1 and TvCyP2, respectively). TvCyP19 localizes to the cytoplasm and to hydrogenosomes. It interacts with transcription factor Myb1, participating in the translocation of the transcription factor to the nucleus [27]. TvCyP19.9 is present in ER membranes and can associate with TvCyP19. Thus, both CyPs could be involved in a putative trafficking pathway [30]. Almost all the PPIases of *T. vaginalis* (except TvFKBP-63) have UniProt annotations indicating their subcellular locations (Table 1). Most of the PPIases are predicted to be present in the cytoplasm and a few in the ER (TvFKBP-15.1 and TvFKBP-15.2) or nucleus (TvCyP14, TvCyP37, TvCyP44, TvCyP63, and TvPar102) (Table 1).

Limited information is available regarding the functions and subcellular locations of PPIases in *E. histolytica*. However, predictions made by UniProt suggest subcellular locations for these proteins. EhCyP18, EhCyP20, EhCyP21, and EhCyP22 may be located in the cytoplasm; EhCyP40 in the ER; EhPar13 in the nucleus; and EhPar13.2 in the cytosol and nucleus. The locations of the remaining *E. histolytica* PPIases could not be predicted (Table 2).

There is little information on the subcellular localization of *G. intestinalis* PPIases. Only certain *Giardia* PPIases, such as GiCyP21 (DH isolate), exhibit membrane localization, and according to UniProt annotations, GiCyP18, GiCyP21, GiFKBP-13, GiFKBP-24, and GiFKBP-38 of the WB isolate have a cytoplasmic presence [29] (Table 3). Only three reports are available on Giardia PPIase localization and functions. In 2017, Ma’ayeh et al. [62] identified PPIases in the *Giardia* secretome from isolates WB and GS; GiCyP18 and GiFKBP-38 were found in both GiCyP21 in WB and GiFKBP-12 in GS. Interaction with the host cells resulted in the secretion of the following five PPIases from both isolates: GiCyP18, GiCyP21, GiFKBP-12, GiFKBP-24, and GiFKBP-38 (Table 3). Moreover, the authors proposed that non-SP secreted proteins, such as GiCyP18 (in WB and GS isolates), which lacks SP, might be released via vesicles. Additionally, GiCyP18 (in the WB isolate), a highly expressed secreted protein [140], has been suggested to play a role in triggering macrophage pyroptosis via TLR4 signaling [60]. However, the detailed role of PPIases in giardiasis remains to be explored.

Among trypanosomatids, *T. cruzi* has been the focus of most PPIase research. TcCyP19 is the most studied CyP and shares 71.9% identity with hCyPA [75]. TcCyP19 is the main CyP expressed and secreted by *T. cruzi* [73,81]. This protein is expressed in all stages of *T. cruzi* [76]. Furthermore, TcCyP19, released by the epimastigote form, inhibits insect antimicrobial peptides, increasing parasite survival [141]. Furthermore, this resistance is related to the mechanism of benznidazole resistance [142]. TcCyP19 is also involved in the modulation of ROS production during infection, promoting *T. cruzi* proliferation [76]. Recently, TcCyP19 has been identified as a promising target of treatment for this disease [143] and seems to be a promising biomarker for the evaluation of trypanocidal therapies and disease diagnosis [144].

**Figure 7:**
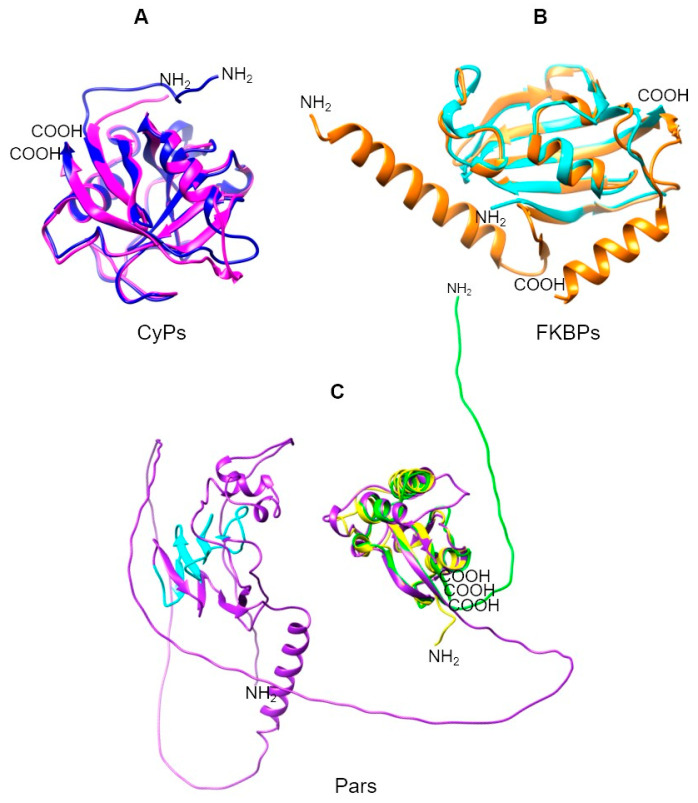
The 3D structures of *T. cruzi* PPIases. (**A**) Overlapping 3D structures of TcCyP21 (PDB: 1XO7) in blue and the extracellular TcCyP19 in pink. (**B**) Overlapping 3D structure of TcMIP (PDB: 1JVW) in orange and a 12 kDa FKBP domain (TcFKBP-12) in cyan. (**C**) Overlapping 3D structure of the following three *T. cruzi* Parvs: TcPar12.6 in yellow, TcPar13 in green, and TcPar45 in purple. The PDB and AlphaFold models were visualized with UCSF Chimera 1.17.1 [64].

Moreover, TcCyP21, a low-abundance protein with an SP (Figure 7A), was identified in a membrane-enriched fraction [145]. TcCyP22, a homolog of mammalian CyPD, localizes to the mitochondria in all three stages of the *T. cruzi* life cycle and is involved in parasite cell death under oxidative stress. In addition, TcCyP21, TcCyP22, TcCyP24, and TcCyP25 are predicted to localize to the mitochondria [77].

Furthermore, the UniProt annotations for TcCyP30 and TcCyP42, two of the four CyPs with elongated regions in their N terminus, indicate that these CyPs exhibit cell membrane localization due to their transmembrane regions. Mitochondrial TcCyP22 is another CyP with this extended region. Thus, these elongations are not random or disordered regions; rather, they could represent specific localization signals that have not been previously reported.

TcFKBP-22 (TcMIP) is the only *T. cruzi* FKBP whose function and localization have been previously described. TcFKBP-22 possesses an SP (Figure 7B) and is secreted by trypomastigotes, playing an important role in the process of host cell entry and invasion [79,146]. Additionally, information on two of the three Pars have been reported (Figure 7C). TcPar12.6 is cytosolic and nonessential for cell proliferation, although the protein is present in all parasite stages [85]. TcPar45 is more nuclear than cytosolic [83] due to its phosphopeptide recognition module (FHA domain). This Par might be involved in various cellular processes, such as signal transduction, protein transport, transcription, protein degradation, and DNA repair [84].

In this review, we infer that the functional PPIases in *T. brucei brucei* are closely related to those in the *T. brucei gambiense* strain (>98.7% identity) and likely present the same location and function in *T. brucei gambiense* strain DAL972. *T. brucei gambiense* CyP19 (TbgCyP19), a hCyPA homolog, is secreted and localized to the cytosol and flagellum [85,87]. Due to this location diversity, this gene product is suggested to have a role in the survival strategy of *Trypanosoma*. TbgCyP21.2 and TbgCyP38 are other secreted CyPs that might be part of protein complexes, considering that TbgCyP38 possesses a predicted TPR motif. Additionally, CyPs lacking an SP could be secreted through microvesicles as an alternative secretory pathway [87]. However, only two of these CyPs have UniProt annotations indicating their subcellular localization. TbgCyP43, which has an elongation in its N terminus, is predicted to localize to the parasite membrane, and TbgCyP58 is predicted to localize to the nucleus due to the presence of an SP and a structural motif for RNA recognition (RRM).

Among the *T. brucei* FKBP proteins, only TbgFKBP-12.3 has been characterized. It is associated with the cytoskeleton and is located in the flagellar pocket, mainly in the bloodstream form of the parasite. TbgFKBP-12.3 contributes to cytokinesis in the bloodstream form and to motility in the procyclic form [88]. Interestingly, only TbgFKBP-48 has been identified as a secreted protein [87]. TbgFKBP-48 contains a predicted TPR, which, like that of TbgCyP38 mentioned above, suggests a potential role in the assembly of protein complexes. Among Pars, only the localization of TbgPar12 (TbgPin1) and TbgPar42 has been previously described [147]. TbgPar12 is localized to the cytosol, and TbPar42 is localized to the nucleus, similarly to their *T. cruzi* homologs, TcPar12.6 and TcPar45, respectively. Furthermore, the association of TbPar42 with cell growth suggests that its function might resemble that of its counterpart Parv in *T. cruzi*.

Experimental evidence regarding the localization and function of PPIases in other trypanosomatids, such as *Leishmania*, remains limited. In the case of *L. major*, the UniProt database predicts that the majority of the PPIases are localized to the cytoplasm (Table 6). Moreover, the discovery that the two crystallized CyPs (LmCyP25 and LmCyP29) of *L. major* are components of the mitoribosome [94] suggests that these CyPs are involved in the *cis*-*trans* isomerization of newly synthesized peptides. This mechanism is similar to that of *E. coli* trigger factor PPIase, which catalyzes the *cis-trans* isomerization of RNAse T1 at the 50S ribosomal subunit [146]. Moreover, the *Vibrio cholerae* trigger factor has a similar interaction with the 50S ribosomal subunit, suggesting its involvement in the *cis-trans* isomerization of novel peptides [148].

Among *L. donovani* CyPs, LdCyP20.4 (also referred to as LdCyP) is a noncytolytic CyP [96] that is released into the cytoplasm and is localized in the ER of the parasite under stress conditions. This translocation pattern suggests a regulatory role during transformation in *L. donovani* [149]. In addition, LdCyP20.4 has a chaperone function that contributes to the disaggregation of adenosine kinase (AK) aggregates in vitro and prevents AK aggregation in vivo [101,150]. Moreover, according to Yau et al., 2010 [100], LdCyP19 (CyP2 identified by MS) and LdCyP38 (also known as LmCyP40) might be implicated in *Leishmania* growth or differentiation. Interestingly, LdCyP38 was identified as a phosphoprotein in amastigotes, and LdFKBP-47 was identified as a phosphoprotein in both stages [150,151,152]. This finding suggests that this post-translational modification can regulate protein activity, location, and interactions in a stage-specific manner. Nonetheless, further research is needed to validate the precise roles of these PPIases.

Among *P. falciparum* CyPs, PfCyP22 and PfCyP19 may be cytosolic CyPs [114]. Additionally, PfCyP22 is localized to the membrane [117], consistent with the UniProt annotation. *P. falciparum* is the parasite with the second most UniProt annotations for subcellular CyP location, following *T. vaginalis*. CyPs are found in the cytoplasm (PfCyP26 and PfCyP32), mitochondria (PfCyP32), and nucleus (PfCyP18.6, PfCyP23, PfCyP53, PfCyP72, PfCyP81, and PfCyP87). Among the *P. vivax* CyPs, only PvCyP65 is suggested to localize to the nucleus (Table 8 and Table 9). The prevalence of the prediction of nuclear localization for these CyPs is intriguing. Notably, PfKBP-35 is the only FKBP with a nuclear prediction in UniProt, which is supported by the data of Kumar et al. (2005) [112], suggesting a role in parasite–nucleosome interactions [153].

The localization of *T. gondii* PPIases is similar. However, studies on the localization and functions of these CyPs are rare. For example, TgCyp18-induced nitric oxide production plays a critical role in inhibiting parasite replication and triggering bradyzoite development [133]. TgCyP20 is a secreted protein that interacts with cysteine–cysteine chemokine receptor 5 (CCR5) and triggers IL-12 production [131]. Interestingly, its PPIase activity is not necessary for the CCR5 interaction but is required for IL-12 induction [154]. *T. gondii* employs a sophisticated strategy of manipulating pro- and anti-inflammatory host cell signaling to promote parasite growth and dissemination while preserving host survival. Furthermore, the UniProt database suggests that TgCyP66.21 and TgCyP66.25 are localized to the nucleus, while TgCyP38 and TgFKBP-38 are found in the membrane. Nonetheless, additional research is required to explore other functions of *T. gondii* PPIases.

There are no experimental studies regarding the localization and function of *Cryptopoisdium spp*. PPIases in parasites. However, in silico predictions show that the PPIases of this parasite are located mainly in the cytosol, plasma membrane, and spliceosomes (Table 11 and Table 12) [29]. PPIases located in the nucleus (CpCyP34 and ChCyP19), spliceosome (CpCyP19, CpCyP89, ChCyP19, and ChCyP89), or nucleolus (CpFKBP-34, CpFKBP-37, ChFKBP-34, and ChFKBP-37) showed similar localization of PPIases found in other parasites (PfCyP87 and TgCyP86) or in humans (PPWD1). It can also be predicted that these proteins may help in the proper folding or formation of spliceosomes, since this process is also inhibited in the presence of CsA [35,40,155].

Although some advances have been made in understanding the localization and functions of PPIases in parasites, much remains unknown. Furthermore, understanding the significance of isoform-specific extensions of PPIases might provide valuable insights into the precise mechanisms of these proteins in parasite biology and pathogenesis, making them attractive targets for further investigation and potential therapeutic interventions.

## 4. Recombinant Expression and Purification of PPIases from Clinically Important Protists

Producing recombinant proteins from protist parasites is often difficult because of the challenges of both the protein expression in the model organism itself and the heterologous expression of recombinant proteins with enzymatic activity [156]. This difficulty arises from the uniqueness of protist protein sequences and the intrinsic complexity of certain proteins [157].

The lack of information about PPIase proteins in many protists has underscored the importance of their recombinant production for molecular and biochemical characterization. Researchers have successfully generated recombinant PPIases with full enzymatic activity through heterologous expression. Various commercial and modified expression vectors can include His or GST tags or solubility tag SUMO (Table 13). *E. coli* is the preferred expression platform; multiple strains have been used, including Rosetta, JM109, XL-Blue, and BL21 (DE3), the latter of which is the most widely used. Most utilized purification methods involve affinity and ion-exchange chromatography, e.g., IMAC, IEX, and GST/GSH Sepharose (Table 13).

The expression of six of the *T. cruzi* CyPs (TcCyP19, TcCyP21, TcCyP25, TcCyP28, TcCyP34, and TcCyP40) was investigated. The TcCyP19 coding sequence was cloned into a pQE30 expression vector and expressed in the M15 *E. coli* strain with 1 mM of IPTG [75]. TcCyP19 and TcCyP40 coding sequences were cloned into a pQE30 plasmid and expressed in XL1 Blue and M15 *E. coli* strains, respectively. Additionally, TcCyP25 and TcCyP34, were cloned into a pRSETA plasmid and expressed in Origami and BL21 DE3 *E. coli* strains, respectively. Furthermore, TcCyP28 gene was cloned into a pET41b plasmid and expressed in the BL21RIL *E. coli* strain. The TcCyP21 coding sequence was cloned into a pET14 vector and expressed in the BL21 PLys *E. coli* strain. The cyclophilins were purified via Immobilized Metal Affinity Chromatography (IMAC) on a Ni-NTA agarose column [78]. Moreover, only one *T. cruzi* FKBP with recombinant expression was reported. The TcMIP gene was cloned into a pGEX-2T expression vector, expressed in the XL1 Blue *E. coli* strain with 0.1 mM IPTG for 5 h, and purified on a Sepharose-glutathione affinity column [79]. *T. cruzi* is the only parasite in which all of the Par proteins were recombinantly produced. The TcPin1 coding sequence was cloned into a pQE30 vector, expressed in the JM109 *E. coli* strain with 0.4 mM IPTG for 6 h at 37 °C, and purified by IMAC. Coding sequences for TcPar14 and TcPar45 were cloned into the pET22b(+) and pET28a vectors, respectively, and expressed in the BL21-CodonPlus (DE3)-RIL *E. coli* strain. Bacterial cultures were grown similarly to TcPin1 and purified by IMAC and Size Exclusion Chromatography (SEC) with a Superdex 75 column [83].

*T. brucei* TbgCyP19 was also cloned into a pQE30 expression vector, expressed in the M15 *E. coli* strain, and purified by IMAC [86]. The TbgPar12 and TbgPar42 coding sequences were cloned into the pET28b vector and expressed in the BL21 (DE3) *E. coli* strain with 0.4 mM IPTG. The proteins were purified by IMAC and SEC using a Superdex 75 column [147].

In *Trichomonas* spp. and *Leishmania* spp., not many PPIase proteins have been recombinantly produced. However, those that have been produced are successfully purified from the soluble fraction, such as TvCyP19 and TvCyP19.9 from *T. vaginalis.* These proteins were the first two recombinantly generated PPIases in *T. vaginalis*. Their coding sequences were cloned into pET vectors modified by Dr. Tai’s group for expression without and with His and GST tags [27,30]. It should be noted that for most of the recombinant proteins of *Leishmania* and *Trichomonas*, short expression times at temperatures of 30–37 °C are sufficient to achieve adequate overexpression of the protein. Methods to purify *T. vaginalis* and *Leishmania* spp. proteins include nickel or GST affinity chromatography (Table 13). Most of the recombinant proteins from these two organisms are cyclophilins, with a range size of 17–20 kDa. No FKBP proteins have been recombinantly produced yet. Only one parvulin-like protein has been recombinantly produced in *Leishmania* spp., namely LmPar13, which was expressed in *E. coli* for 20 h at 18 °C and purified by nickel affinity chromatography [101].

Most of the cyclophilins of *P. falciparum* PPIases have been recombinantly expressed and purified. PfCyP19 and PfCyP22 have been extensively characterized. Their coding sequences were cloned into pET-3a and pET22b+ vectors, respectively, and purified using IMAC. However, some difficulties in expression of the other *P. falciparum* cyclophilins have been reported. One of the alternatives to express them is to amplify and express only the CyP domain (PfCyP32 and PfCyP53). However, PfCyP32 was only obtained from the insoluble fraction as inclusion bodies [108,113,119]. In contrast, PfKBP35 was successfully cloned in pMAL-c2X and pSUMO vectors; expressed in the BL21 *E. coli* strain; and purified by IMAC, GFC, and AC. However, expression and purification of FKBP25.6 are still lacking [111,112,123,153,160]. The opposite case is true for *P. vivax*, since only its two of its FKBP PPIases have been recombinantly expressed [122,159].

TgCyP18 and TgCyP23 of *T. gondii* cyclophilins were successfully expressed using the pET28a vector in the BL21(DE3) *E. coli* strain with 0.5 mM IPTG for 16 h at 22 °C, followed by purification by IMAC [132]. The dual PPIase TgFCBP57 and its individual domains have also been successfully produced; they were cloned into the pET15b vector, expressed in the BL21(DE3) *E. coli* strain, and purified by IMAC [130].

In *G. intestinalis,* three PPIases, namely GiCyP1, GiCyP18, and GiCyP19, and one FKBP, namely GiFKBP12, have been successfully expressed as recombinant proteins. All of them were expressed in *E. coli* and recovered as soluble proteins [60,65,67].

To date, there are no reports of expression or purification of *C. parvum* or *C. hominis* PPIases. However, two crystallized Cyps, namely CpCyP18 and CpCyP19, are currently available in the PDB. Each has two structures, namely CpCyP18 alone or in complex with cyclosporin A and CpCyP19 with or without the ala-pro dipeptide. Both CpCys were expressed in *E. coli* [58]. However, no further information on their purification was provided.

As observed, PPIases from *E. histolytica*, *G. intestinalis*, and *P. vivax* have undergone the least heterologous production and study among the clinically important parasites. In contrast, more than 50% of recombinant PPIases are from *T. cruzi* and *P. falciparum* (Table 13). These recombinant PPIases (rPPIases) from parasites have been produced for structural analysis, biological characterization, antibody production for further studies, and research on their potential as therapeutic targets.

The primary focus of rPPIase production has been on CyPs rather than on FKBPs and Pars. Only five FKBPs have been produced, namely *G. intestinalis* (GiFKBP-12), *T. cruzi* (TcFKBP-22), *P. falciparum* (PfFKBP-35), *P. vivax* (PvFKBP34), and *T. gondii* (TgFCBP-57). Only six Pars have been recombinantly produced from trypanosomatids, namely *T. cruzi* (TcPar12.6, TcPar14, and TcPar45), *T. brucei* (TbgPar12 and TbPar42), and *L. major* (LmPar13). The scant production of recombinant FKBPs can be attributed to the sequence complexity of some of these proteins. Furthermore, FKBPs and Pars are relatively new discoveries compared to CyPs, which is another reason they are only beginning to be studied in protist parasites (Table 13).

Notably, most of the protozoan rPPIases have been obtained in the soluble fraction, except for TgCyP18, which was purified from inclusion bodies but not used in activity assays [131]. These soluble proteins have molecular weights of between 18 and 30 kDa (Table 13). Difficulties in obtaining large recombinant proteins were evident with certain CyPs from *P. falciparum*, such as PfCyP32, PfCyP72, PfCyP81, and PfCyP87, which cannot be cloned or expressed in *E. coli*. Consequently, only the CLD was expressed, except for PfCyP81, which could not be produced.

Interestingly, many rPPIases from parasites have been expressed in the soluble fraction, in contrast to the general challenges faced in obtaining recombinant proteins from protist parasites. Typically, it is estimated that only 30–50% of parasite proteins are heterologously expressed, and an even smaller fraction of those proteins is successfully purified [108].

## 5. Assays on the Activity of PPIases from Clinically Important Protists

Most of the activity assays conducted for the recombinant PPIases discussed in this review are based on a spectrophotometric assay proposed by Fischer (1984) [161] and modified by Kofron et al. (1991) [162]. The Kofron assay is commonly used to evaluate the *cis-trans* isomerization of chromogenic peptide *N*-suc-APPF-pNA by PPIases via a chymotrypsin-coupled method. Additionally, modifications have been made to the chromogenic substrate sequence to assess the enzymatic affinity of PPIases. For example, succinyl-Ala-Leu-Pro-Phe-p-nitroanilide has been widely used for analysis of FKBP PPIase activity [163]. In the case of Pars from *T. brucei*, activity was evaluated using phosphorylated peptide SSYFSG[p]TPLEDDSD, as Pars are known to exhibit activity on phosphorylated peptides [147]. A protease-free variant of the Kofron assay has also been used to evaluate PPIase activity. For instance, in the case of *T. cruzi* Pars, a succinyl-Ala-Glu-Pro-Phe-p-nitroanilide substrate was used that included a negatively charged glutamyl instead of a positively charged alanine, modifying the classical substrate of the assay [83,85].

Several CyPs, such as PfCyP19 and PfCyP22 from *P. falciparum,* LmCyP19 from *L. major,* and TgCyP23 from *T. gondii*, have demonstrated high levels of activity comparable to those of hCyPA (*Kcat/Km* = 4.9 × 10^6^ M^−1^s^−1^) [113,132]. Moreover, among the recombinant CyPs from protist parasites, TvCyP19 and TvCyP19.9 from *T. vaginalis* exhibited the lowest PPIase activity. Their activity is lower than that hCyPA [27,30,132], which could be attributed to differing substrate affinities. These data suggest that these TvCyPs might exhibit different activities on other substrates (Table 13). Moreover, it is important to note that some CyPs showed no PPIase activity, such as several *P. falciparum* recombinant CyPs (PfCyP18.6, PfCyP23, PfCyP25, PfCyP26, PfCyP32 CLD, and PfCyP25 CLD). However, the activities of these proteins were evaluated using two different methods, namely the classical Kofron assay and RNAse T refolding. One possible reason for the lack of enzymatic activity might be the absence of H^126^ in the catalytic site, an aa residue considered crucial for binding to CsA in hCyP18. Notably, some hCyPs lack PPIase activity while still retaining their chaperone role. Hence, it is plausible that both functions are not universally associated with all PPIases [119].

Regarding FKBP activity, only three out of the six recombinant protist FKBPs produced thus far have been evaluated. Among these, the *P. falciparum* and *P. vivax* FKBPs, PfFKBP-35 and PvFKBP-34, exhibited similar activities, both of which were greater than the activity of *T. cruzi* TcFKBP-22 (Table 13). In contrast, PvFKBP-25 showed no PPIase activity, which could be attributed to mutations in the active site similar to those in *T. gondii* CyPs. These data suggest that this FKBP in *P. falciparum* might differ from the others [122]. Notably, protist FKBPs generally exhibit lower activity than CyPs. This difference was also observed for hFKBP, which has 25 times lower activity than hCyP. This significant difference in activity could be due to the varying affinities of FKBPs for the substrate used in the Kofron assay [164].

Only Pars from *T. cruzi* have been analyzed using two different substrates. Specifically, rTcPar14 and rTcPar45 demonstrated very high affinities for the succinyl-Ala-Arg-Pro-Phe-NH-Np substrate, while their affinities were minimal or negligible for substrates lacking arginine immediately preceding proline [83]. Conversely, TcPar12.6 exhibited a greater affinity for the Ala-Glu-Pro-Phe-p-nitroanilide substrate than for the other tested substrates [82].

These data are significant because they highlight the importance of considering cases where recombinant PPIases exhibit little or no activity. These proteins might possess distinct functions or higher activity levels with different substrates. Therefore, identifying specific substrates for parasite PPIases represents a vital area of research that deserves further development.

## 6. Inhibition Assays of rPPIases from Protozoan Parasites

The importance of PPIase inhibitors has been highlighted since the discovery of CyPs that bind CsA, a molecule with immunosuppressive activity [11]. The antiparasitic activity of CsA is more strongly associated with calcineurin inhibition than PPIase inhibition [165]. To fully understand the mechanism by which CsA inhibits infection, it is necessary to identify the parasite CsA receptor [132]. An important part of parasite rPPIase studies is identifying whether known inhibitors (CsA, FK506, and rapamycin) or new inhibitors inhibit multiple biological processes in which PPIases are involved.

For example, the recombinant CyPs that showed high sensitivity to CsA were TvCyP19 and TvCyP19.9 from *T. vaginalis*, EhCyP18 from *E. histolytica*, PfCyP19 and PfCyP22 from *P. falciparum*, and TgCyP23 from *T. gondii* in the nanomolar range (IC_50_= 0.6–10 nM), comparable to the inhibition of hCyPA (6.6 nM) [113]. Intermediate sensitivity to CsA (IC_50_ 13–31 nM) was observed for GiCyP19 from *G. intestinalis* and TcCyP19, TcCyP21, TcCyP25, and TcCyP28 from *T. cruzi*. However, the lowest sensitivity to CsA (IC_50_ of160 nM) was determined for TcCyP35.1 and TcCyP38 from *T. cruzi* and LmCyP38 and LdCyP20.4 from *Leishmania* (Table 13). These values are comparable to those of hCyP40 [166]. In contrast, the dual PPIase TgFCBP-57 from *T gondii* required high concentrations of CsA for inhibition (Table 13).

Moreover, the competitive inhibition constant (*K_i_*) of PPIases has been reported only for LmCyP19 (*K_i_* =5.2 nM) from *L. major* and PfCyP19 from *P. falciparum* (*K_i_* =3.3–14.4 nM), which showed similar affinities for CsA [98,113]. These values are also comparable to the *K_i_* value of a mammalian CyP (*K_i_ =* 3 nM) [150]. Interestingly, not all recombinant CyPs have detectable enzymatic activity. The *P. falciparum* CyPs, which have molecular weights of between 18.6 and 53 kDa, do not (Table 13). Moreover, despite their strong in vitro inhibitory effects on *P. falciparum* CyPs (Table 13), the CsA derivatives did not exhibit significant antimalarial activity in in vivo tests, unlike CsA [113]. Among the nine recombinant CyPs, only one exhibited high sensitivity to CsA derivatives (Table 13). In addition, no immunosuppressive CsA derivatives demonstrated IC_50_ values comparable to those of CsA in inhibition assays using recombinant *T. cruzi* CyPs, except for TcCyP35.1 and TcCyP38, which required high concentrations of inhibitors [78]. Variations in affinities between PPIases and their inhibitors, such as TgCyP18.4 and TgCyP23, have been attributed to changes in crucial binding-site residues. These alterations influence the affinities of these PPIases for CsA [132].

Inhibition assays with FK506 and the four FKBs showed IC_50_ values in the nanomolar range (70–410 nM) (Table 13). Other inhibitors tested on FKBPs, such as L-685-818, rapamycin, and D44, also had IC_50_ values in the nanomolar range (Table 13). Notably, the double inhibition of TgFCBP-57 from *T. gondii* by CsA and FK506 identified this protein as an FCBP, a protein with both CLD and FKBP domains. It is the only FCBP from protozoa that has been recombinantly produced thus far [130]. These inhibitors also reduced parasitic infection and growth [73,167].

Interestingly, inhibitor D44 selectively targeted PfFKBP-35 through its PPIase activity and inhibited *P. falciparum* growth [160]. Notably, this PPIase can inhibit calcineurin independently of the presence of the inhibitor [130]. There are no reports on inhibition assays of protozoan Pars, although the juglone inhibitor inhibits this type of PPIase [8]. Since a unique feature of Par is its binding to phosphopeptides using a positively charged surface, inhibitors of this protein may require negatively charged substituents [168].

Finding specific and relevant inhibitors for PPIases is challenging due to several factors, including the superficiality of their binding sites. This characteristic makes it difficult to create small-molecule inhibitors that can bind to enzymes with high affinity. In general, PPIases have structurally conserved binding sites across different families, further complicating the search for inhibitors [168]. Finally, inhibiting PPIase activity may not always affect parasite infection, as evidenced in some studies.

## 7. Biotechnological Applications of Protozoan PPIases

**Figure 8:**
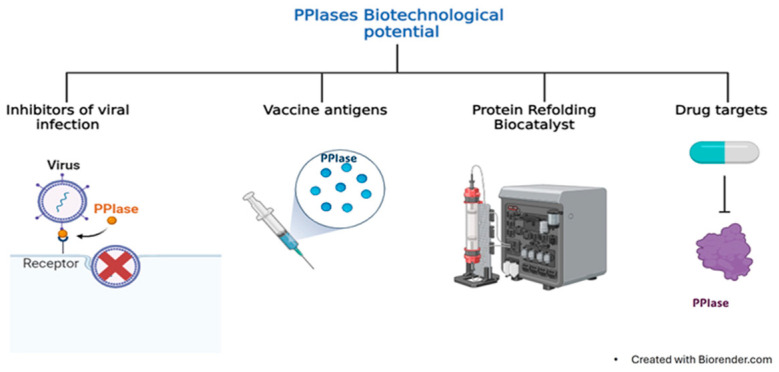
Biotechnological applications of protozoan PPIases.

The important roles of protozoan parasite PPIases in protein folding, sexual differentiation, virulence, and immunomodulation make these proteins potential drug targets. Many parasite PPIases play important roles as virulence factors or are important in parasite life cycles. Therefore, these PPIases have potential biomedical and biotechnological applications (Figure 8).

These PPIases are also considered potential inhibitors of viral infections. For example, TgCyP18 from *T. gondii* was found to be an inhibitor of HIV-1 cell fusion and cell-free viral infection. This protein binds to human immunodeficiency virus (HIV) coreceptor CCR5 and inhibits viral fusion and infection of T cells and macrophages. Importantly, such findings may lead to new anti-HIV drugs [169,170]. Moreover, TgCyP18 has the potential for use as a vaccine antigen. It has been tested in combination with vehicle and BCG adjuvant in a vaccine against *T. gondii*. This vaccine antigen was found to be highly immunogenic and showed good protection against *T. gondii* infection in BALB/c mice [171,172].

No reports on the use of PPIases in any study related to *C. parvum* or *C. hominis* were found in the literature. However, it should be noted that PPIases from *Cryptosporidium* spp., as well as their inhibitors, are an important case study as molecular markers, vaccine antigens, or therapeutic molecules and in the search for new inhibitors of PPIase activity, as they are closely related to cellular and infectivity processes, as observed in other parasites. The only study of *Cryptosporidium* related to PPIase inhibitors was reported by Perkins et al. (1998) [173]. They found that both cyclosporin and some analogs (SDZ 033-243 and SDZ PSC-833) inhibited the growth of *C. parvum.* However, further studies are needed to relate this effect to PPIases.

PPIases have also assisted in the in vitro refolding of denatured proteins. One example is the refolding of human creatine kinase, a protein with many prolines in its sequence. This enzyme was denatured in 6 M urea and refolded in the absence or presence of human PPIase. The results showed that PPIase accelerated the slow phase of refolding, and the enzyme became active at the end of the refolding process. This highlighted the *cis-trans* isomerization of its prolines as the critical step in the refolding of human creatine kinase [174]. However, no research has been conducted on assisted refolding by parasite PPIases. Specifically, it would be interesting to analyze them in the refolding of recombinant proteins from the same parasite expressed as inclusion bodies that contain many prolines, such as TSA-1 of *T. cruzi*, a promising antigen for the development of a therapeutic vaccine against Chagas disease [175].

## 8. Conclusions

PPIases are found in large numbers in most clinically important protozoans. However, these enzymes have not been fully studied, possibly because many of them have complex structures. Notably, PPIases play important roles as chaperones, participating in various parasite functions. Thus, several PPIases are considered virulence factors, suggesting that they are potential targets for therapeutic inhibition and candidate vaccine antigens against parasitic infections. Therefore, the recombinant production of protozoan PPIases is an important and necessary tool to expand the biological and biotechnological information on these enzymes and determine their potential as therapeutic targets. Interestingly, PPIases from different parasites are often recombinantly produced in a soluble form and with catalytic activity. These characteristics endow these proteins with great potential for use in different biotechnological applications.

## Figures and Tables

**Table 2:** Peptidyl-prolyl cis-trans isomerase repertoire from *Entamoeba histolytica* ^1,2^.

UniProt	AmoebaDB	NCBI	PPIase Name	Localization ^3^	References
C4LYX1	EHI_117870	XP_656069.1	EhCyP10		
O15729	EHI_125840	XP_656494.1	EhCyP18 (EhCyP)	Cytoplasm	[29,45]
C4M7U6	EHI_020340	XP_654585.1	EhCyP20	Cytoplasm	[29]
C4M525	EHI_128100	XP_648283.1	EhCyP21	Cytoplasm	[29]
C4M942	EHI_083580	XP_654418.1	EhCyP22	Cytoplasm	[29]
C4M2J5	EHI_054760	XP_654797.2	EhCyP40	Nucleus	[29]
C4LTN0	EHI_012390	XP_655852.2	EhFKBP18	ER	[29]
C4M276	EHI_180160	XP_653822.1	EhFKBP29		
C4LTA4	EHI_044850	XP_657211.1	EhFKBP35		
B1N302	EHI_051870	XP_001913568.1	EhFKBP43		
C4LUU9	EHI_178850	XP_656239.1	EhFKBP46		
C4M181	EHI_188070	XP_653673.2	EhPar13	Cytoplasm and nucleus	[29]
C4LT92	EHI_044730	XP_657226.1	EhPar13.25	Nucleus	[29]

^1^ Isolate *E. histolytica* HM1-IMSS. ^2^ Previously reported names are presented in parentheses. ^3^ The localization and functions of PPIases were taken from the cited references or from the UniProt database and were predicted by the Gene Ontology Consortium [29]. ER: endoplasmic reticulum. Blank spaces: data not reported.

**Table 5:** Peptidyl-prolyl cis-trans isomerase repertoire from *Trypanosoma brucei gambiense* ^1^.

UniProt	TriTrypDB	NCBI	PPIase Name ^2^	Localization ^3^	Function ^3^	References
D0A5M6	Tbg972.11.920(CYPA)	XP_011779241.1	TbgCyP19 (TbgCyPA)	Cytoplasm, flagellum, and extracellular space		[85,86]
C9ZYX4	Tbg972.9.6990	XP_011776889.1	TbgCyP20.3			
D0A8E1	Tbg972.11.10610	XP_011780206.1	TbgCyP20.5			
C9ZIV0	Tbg.972.2.170	XP_011771617.1	TbgCyP21.1			
C9ZT99	Tbg972.7.5450	XP_011774914.1	TbgCyP21.2	Extracellular space		[86]
C9ZWH7	Tbg972.8.7100	XP_011776042.1	TbgCyP21.4			
C9ZRQ0	Tbg972.7.160	XP_011774319.1	TbgCyP24			
C9ZSQ5	Tbg972.7.3760	XP_011774720.1	TbgCyP25.55			
C9ZNS2	Tbg972.5.1880	XP_011773337.1	TbgCyP25.56			
C9ZWA7	Tbg972.8.6340	XP_011775972.1	TbgCyP27.1			
C9ZXF5	Tbg972.9.1740	XP_011776370.1	TbgCyP27.4			
C9ZQE6	Tbg972.6.1040	XP_011773911.1	TbgCyP29			
C9ZVY5	Tbg972.8.5140	XP_011775850.1	TbgCyP30			
C9ZUX8	Tbg972.8.1650	XP_011775493.1	TbgCyP33			
C9ZYI8	Tbg972.9.5630	XP_011776753.1	TbgCyP38	Extracellular space		[86]
C9ZZI1	Tbg972.9.9060	XP_011777096.1	TbgCyP43	Membrane		[29]
C9ZIB2	Tbg972.1.930	XP_011771345.1	TbgCyP46			
C9ZPQ4	Tbg972.5.5220	XP_011773669.1	TbgCyP58	Nucleus		[29]
C9ZZU0	Tbg972.10.15980	XP_011778762.1	TbgCyP100			
D0A2I5	Tbg972.10.5640	XP_011777743.1	TbgFKBP12			
C9ZSQ4	Tbg972.7.3750	XP_011774719.1	TbgFKBP12.3 (TbgFKBP12)	Flagellar pocket	Motility and cytokinesis	[87]
D0A0P0	Tbg972.10.19020(MIP)	XP_011779062.1	TbgFKBP21			
D0A0P1	Tbg972.10.19030	XP_011779063.1	TbgFKBP36			
D0A0V5	Tbg972.10.19710	XP_011779127.1	TbgFKBP48	Extracellular space		[86]
D0A6H9	Tbg972.11.3980	XP_011779544.1	TbgFKBP92			
C9ZUI9	Tbg972.8.300(Pin1)	XP_011775354.1	TbgPar12 (TbgPin1)	Cytoplasm		[88]
C9ZKX9	Tbg972.3.3260	XP_011772278.1	TbgPar13 (TbgPar14)			[88]
C9ZRL7	Tbg972.7.2770(Par45)	XP_011774600.1	TbgPar42	Nucleus	Cell growth	[88]

^1^ Isolate: *Trypanosoma brucei gambiense* DAL972. ^2^ Previously reported PPIase names are presented in parentheses. ^3^ The localization and functions of PPIases were taken from the cited references or from the UniProt database and were predicted by the Gene Ontology Consortium [29]. Blank spaces: data not reported.

**Table 6:** Peptidyl-prolyl cis-trans isomerase repertoire from *Leishmania major* ^1,2^.

UniProt	TriTrypDB	NCBI	PDB	PPIase Name	Localization ^3^	References
O02614	LmjF.25.0910 (CYPA)	XP_001683845.1		LmCyP19 (LmaCyP1)	Cilium, Cytoplasm, and Nucleus	[29,94,95]
Q4QJ67	LmjF.06.0120 (CYP2)	XP_001680781.1		LmCyP20.3 (LmaCyP2)	Cytoplasm	[29,96]
Q4QBG3	LmjF.23.0125 (CyP3)	XP_001683335.1		LmCyP20.4 (LmaCyP3)	Nucleus	[29,96]
Q4Q424	LmjF.33.1630 (CYP4)	XP_001685924.1		LmCyP24 (LmaCyP4)	Cytoplasm	[29,96]
Q4Q6Q9	LmjF.31.0050 (CYP5)	XP_001684989.1		LmCyP24.6 (LmaCyP5)	Cytoplasm	[29,96]
Q4QBK2	LmjF.22.1450 (CYP6)	XP_001683296.1	7AIH	LmCyP25 (LmaCyP6)	Cilium, cytoplasm, and nucleus	[29,94,96]
E9AFI5	LmjF.35.3610 (CYP7)	XP_003722755.1		LmCyP26 (LmaCyP7)	Cilium, cytoplasm, and nucleus	[29,96]
Q4QAK0	LmjF.24.1315 (CYP8)	XP_001683648.1		LmCyP26.5 (LmaCyP8)	Cytoplasm	[29,96]
Q4Q7V7	LmjF.30.0020 (CYP9)	XP_001684591.1		LmCyP27 (LmaCyP9)	Axoneme and cytoplasm	[29,96]
Q4Q1A6	LmjF.36.3130 (CYP10)	XP_001686892.1	7AM2	LmCyP29 (LmaCyP10)	Cytoplasm	[29,94,96,97]
Q4QBH1	LmjF.23.0050 (CYP11)	XP_001683327.1	2HQJ	LmCyP32 (LmaCyP11)	Cytoplasm and nucleolus	[29,61,96]
E9AC11	LmjF.01.0220 (CYP12)	XP_003721542.1		LmCyP36 (LmaCyP12)	Axoneme and cytoplasm	[29,96]
E9AFV2	LmjF.35.4770 (CYP40)	XP_003722872.1		LmCyP38 (LmaCyp40)	Cytoplasm	[29,96]
Q4QEP7	LmjF.16.1200 (CYP13)	XP_001682201.1		LmCyP39 (LmaCyP13)	Axoneme and cytoplasm	[29,96]
E9AEZ3	LmjF.35.1720 (CYP14)	XP_003722563.1		LmCyP48 (LmaCyP14)	Cytoplasm and membranes	[29,96]
Q4QCV2	LmjF.20.0940 (CYP15)	XP_001682846.1		LmCyP49 LmaCyP15		[96]
Q4QDV4	LmjF.18.0880 (CYP16)	XP_001682494.1		LmCyP108 LmaCyP16	Nucleoplasm	[29,96]
Q4QBK4	LmjF.22.1430	XP_001683294.1		LmFKBP-11.8 (maFKBPL1)	Axoneme and cytoplasm	[29,96]
Q4Q255	LmjF.36.0230	XP_001686593.1		LmFKBP-11.9 (LmaFKBPL2)		[96]
Q4QHC5	LmjF.10.0890	XP_001681423.1		LmFKBP-17.3 (LmaFKBPL3)		[96]
Q4QDB9	LmjF.19.0970	XP_001682679.1		LmFKBP-23 (LmaFKBPL4)		[96]
Q4QD56	LmjF.19.1530	XP_001682742.1		LmFKBP-48 (LmaFKBPL5)		[96]
Q4QII4	LmjF.07.1030 (PIN1)	XP_001681014.1		LmPar13 (LmaPPICL1) (LmPIN1)	Cytosol and nucleus	[29,96,98]
Q4QBU3	LmjF.22.0530 (PAR45)	XP_001683205.1		LmPar47 (LmaPPICL2)	Nucleus	[29,96]

^1^ Isolate: *Leishmania major* Friedlin. ^2^ Previously reported PPIAse names are presented in parentheses. ^3^ The localization and functions of PPIases are taken from the cited references cited or predicted by the Gene Ontology Consortium [29] in the UniProt database. Blank spaces: no reported data.

**Table 7:** Peptidyl-prolyl cis-trans isomerase repertoire from *Leishmania donovani* ^1,2^.

UniProt	TriTrypDB	NCBI	PDB	PPIase Name	Localization ^3^	Function ^3^	References
E9BHJ8	LdBPK_250940.1 (CYPA)	XP_003861424.1		LdCyP19			
A0A3S7WXE3	LdBPK_230140.1	XP_003860915.1		LdCyP20.3			
Q9U9R3	LdBPK_060120.1	XP_003858320.1	2HAQ	LdCyP20.4 (LdCyP)	Cytoplasm and ER	Disaggregation and aggregation prevention	[99,100,101,102,103]
A0A3S7X410	LdBPK_310060.1	XP_003863096.1		LdCyP24			
A0A3Q8ICB3	LdBPK_221300.1	XP_003860876.1		LdCyP25			
E9BSN7	LdBPK_353660.1	XP_003864946.1		LdCyP26			
E9BGZ8	LdBPK_241350.1	XP_003861226.1		LdCyP27			
A0A3S7X325	LdBPK_300020.1	XP_003862718.1		LdCyP28			
E9BQA4	LdBPK_331730.1	XP_003863999.1		LdCyP28.6	Membrane		[100]
E9BU37	LdBPK_363280.1	XP_003865443.1		LdCyP29			
E9BG26 A0A504XWA0	LdBPK_230060.1	XP_003860907.1		LdCyP32			
A0A451EJ79	LdBPK_010220.1	XP_003857835.1		LdCyP36			
A0A3Q8IIG9	LdBPK_354830.1	XP_003865060.1		LdCyP38.4 (LdCyP40)			[96]
A0A504WZ51	LdBPK_161250.1	XP_003859812.1		LdCyP39			
E9BS46	LdBPK_351710.1 (CYP14)	XP_003864755.1		LdCyP48.5	Membrane		[29]
E9BEP2	LdBPK_200950.1	XP_003860435.1		LdCyP49			
E9BDR8	LdBPK_180880.1	XP_003860101.1		LdCyP108			
E9BFZ3	LdBPK_221280.1	XP_003860874.1		LdFKBP11.8			
E9BT84	LdBPK_360250.1	XP_003865142.1		LdFKBP11.9			
E9BAD9	LdBPK_100940.1	XP_003858930.1		LdFKBP17			
E9BE85	LdBPK_190920.1	XP_003860268.1		LdFKBP22			
E9BEE5	LdBPK_191560.1	XP_003860328.1		LdFKBP47			
E9B9B2	LdBPK_071180.1	XP_003858557.1		LdPar12			
E9BFR0	LdBPK_220410.1	XP_003860791.1		LdPar17			

^1^ Isolate: *Leishmania donovani* BPK282A1. ^2^ Previously reported PPIAse names are presented in parentheses. ^3^ The localization and functions of PPIases are taken from the cited references or predicted by the Gene Ontology Consortium [29] in the UniProt database. ER: endoplasmic reticulum. Blank spaces: no reported data.

**Table 11:** Peptidyl-prolyl cis-trans isomerase repertoire from *Cryptosporidium parvum* ^1^.

UniProt	CryptoDB	NCBI	PDB	PPIase Name	Localization ^2^	Reference
A3FQA7	cgd2_4120	XP_001388243.1	2PLU 2POY	CpCyP18	Cytosol and plasma membrane	[29,61]
Q9Y0F5	cgd2_1660	XP_001388204.1	2POE 2QER	CpCyP19	Spliceosome	[29,61]
F0X4J7	cgd5_3350	XP_001388285.1		CpCyP21.1	Cytosol and plasma membrane	[29]
Q5CW56	cgd8_1560	XP_627063.1		CpCyP21.2	Cytosol and plasma membrane	[29]
Q5CSY2	cgd1_870	XP_627935.1		CpCyP23	Cytosol and plasma membrane	[29]
K9ME21	cgd8_2350	XP_001388428.1		CpCyP34	Nucleus	[29]
Q5CYY7	cgd7_520	XP_628243.1		CpCyP89	Spliceosome	[29]
Q5CZ15	cgd7_210	XP_628215.1		CpFKBP-34	Nucleolus	[29]
Q5CX33	cgd6_2690	XP_627621.1		CpFKBP-37	Nucleolus	[29]

^1^ Isolate: *Cryptosporidium parvum* IOWA II. ^2^ The localization and functions of PPIases were taken from the cited references or from the UniProt database and were predicted by the Gene Ontology Consortium [29]. Blank spaces: data not reported.

**Table 12:** Peptidyl-prolyl cis-trans isomerase repertoire from *Cryptosporidium hominis* ^1^.

UniProt	CryptoDB	NCBI	PPIase Name	Localization ^2^	Reference
A0A0S4TC12	Chro.20441	XP_667665.1	ChCyp18.4 ChCyP18	Cytosol and plasma membrane	[29,138]
A0A0S4TBF4	Chro.20180	XP_667461.1	ChCyp18.9 ChCyP19	Spliceosome	[29,138]
A0A0S4TFY9	Chro.50038	XP_665956.1	ChCyp17.9 ChCyP21	Cytosol and plasma membrane	[29,138]
A0A0S4TJX7	Chro.80184	XP_665525.1	ChCyP21.2	Cytosol and plasma membrane	[29,138]
A0A0S4TA19	Chro.10107	XP_667336.1	ChCyp22.9 ChCyP23	Cytosol and plasma membrane	[29,138]
A0A0S4TLJ2	Chro.80276	XP_666493.1	ChCyp34.5 ChCyP40	Nucleus	[29,138]
A0A0S4THZ0	Chro.70067	XP_666650.1	ChCyp88.9 ChCyP89	Spliceosome	[29,138]
A0A0S4TJS1	Chro.70034	XP_668351.1	ChFKBP-34	Nucleolus	[29]
A0A0S4TGW0	Chro.60310	XP_667889.1	ChFKBP-37	Nucleolus	[29]

^1^ Isolate: *Cryptosporidium hominis* TU502. ^2^ The localization and functions of PPIases were taken from the cited references or from the UniProt database and were predicted by the Gene Ontology Consortium [29]. Blank spaces: data not reported.

**Table 13:** Recombinant PPIases from protozoan parasites expressed in *E. coli* ^1,2^.

Parasite	PPIase	UniProt	kDa	pI	Expression System		Purification	Catalytic Efficiency ^3^	Inhibition		References
					Strain	Vector		*k_cat_/K_m_*	Inhibitor	IC_50_ nM ^4^	
*T. cruzi*	TcCyP19	Q9U664	18.9	8.4	M15, XL1Blue	pQE30	IMAC		CsA H-7-94 F-7-62 MeVal-4	14.4-18.4 12.5 13.3 15.3	[75,78,81]
	TcCyP21	Q4DPB9	21.1	9.1	BL21 pLysS	pET14	IMAC		CsA H-7-94 F-7-62 MeVal-4	28.7 23.6 25.2 30.0	[73,78]
	TcCyP25	Q9NAT5	25.6	8.5	Origami	pRSETA	IMAC		CsA H-7-94 F-7-62 MeVal-4	31.7 17.2 17.8 30.0	[73,78]
	TcCyP28	O76990	28.4	9.7	BL21 RIL	pET41b	IMAC		CsA H-7-94 F-7-62 MeVal-4	13.1 9.2 10.1 13.5	[73,78]
	TcCyP34	K2NAL4	33.4	9.0	BL21(DE3)	pRSETA	IMAC		CsA H-7-94 F-7-62 MeVal-4	>200 ^5^	[73,78]
	TcCyP38 (TcCyP40)	Q6V7K6	38.4	5.7	M15	pQE30	IMAC		CsA H-7-94 F-7-62 MeVal-4	>200 ^5^	[73]
*T. brucei*	TbgCyP19 (TbCypA)	D0A5M6	18.7	8.3	M15	pQE30	IMAC				[86]
*T. vaginalis*	TvCyP19 (TvCyP1)	A2DT06	19	7.7	BL21	pET32a	IMAC, IEX, AC	7.1 μM^−1^s^−1^ 4.0 μM^−1^s^−1^	CsA	7.5	[27,32]
	TvCyP19.9 (TvCyP2)	A2DLL4	20.0	9.1	BL21	pET, pGEX2t pET29b	IMAC, IEX, AC	4.5 μM^−1^s^−1^			[30,31]
*L. major*	LmaCyP19	O02614	19.0	7.7	M15	pQE30, pREP4 pET14b, pTYB1 pGEX4T-3	IMAC, HIC, AC	1.5 × 10^6^ M^−1^s^−1^ 2.6 × 10^6^ M^−1^s^−1^	CsA	*Ki* = 0.5 ^3^	[95,98]
	LmaCyP38 (LmaCyp40)	E9AFV2	38.4	5.6	BL21	pGEX-5X-Strep	AC				[100]
*L. donovani*	LdCyP20.4 (LdCyP)	Q9U9R3	17.7	6.9	BL21 pLysS	pET3a, pQE32	IMAC				[96,97,102,103]
*T. gondii*	TgCyP18	A0A125YZ79	18.3	6.9	BL21	pET28a	IMAC, AC, SEC, RPC	1.0 × 10^4^ M^−1^s^−1^			[132]
	TgCyP20	S8F7V1	19.6	6.0			AC				[131,134]
	TgCyP23	A0A125YL73	22.9	7.0	BL21	pET28a	IMAC, SEC	3.8 × 10^6^ M^−1^s^−1^			[132]
*P. falciparum*	PfCyP19 (PfCyP19A)	Q76NN7	19.0	8.2	BL21	pET-3a, pET22b+	IMAC	6.3 × 10^6^ M^−1^s^−1^ 1.2 × 10^7^ M^−1^s^−1^	CsA CsC CsD Rapamycin FK506	10 581 238 >5000 >10,000	[108,110,113,114,119]
	PfCyP22 (PfCyP19B)	Q8IIK8	22.0	7.1	BL21	pET22b+	IMAC	2.3 × 10^6^ M^−1^s^−1^ 5.7 × 10^6^ M^−1^s^−1^	CsA	10	[108,113,114,115,117]
	PfCyP18.6 (PfCyP19C)	Q8IIK3	18.6	5.9	BL21	pET22b+	IMAC				[108,119]
	PfCyP23	Q8I3I0	23.2	5.3	BL21	pET22b+	IMAC				[108,119]
	PfCyP25 (PfCyP24)	Q8I6S4	24.9	6.7	BL21	pET22b+	IMAC				[108,119,120,122]
	PfCyP26	Q8I621	26.4	8.5	BL21	pET22b+	IMAC				[108,119]
	PfCyP32	Q8I5Q4	32.3	9.8	Rosetta	pET22b+	IMAC				[108,119]
	PfCyP53 (PfCyP52)	Q8ILM0	52.7	7.0	BL21	pET22b+	IMAC				[108,119]
*E. histolytica*	EhCyP18 (EhCyP)	O15729	18.1		XL1Blue	pTrcHis A	IMAC		CsA	10	[45]
*G. intestinalis*	GiCyP19 (GiCyP1)		19.0		BL21	pGEX 4T-1	AC		CsA	500	[65]
	GiCyP18	A8BC67	18.0	8.4	BL21	pColdI	IMAC				[60]
*T. cruzi*	TcFKBP22 (TcMIP)	Q09734	22.1	6.8	XL1 Blue	pGEX-2T	AC	0.745 M^−1^s^−1^	FK506	410	[79,158]
*T. gondii*	TgFCBP57	Q4VKI5	57.2	5.5	BL21(DE3)	pET15b	IMAC		FK506 CsA	70 750	[130]
*P. falciparum*	PfFKBP35	Q8I4V8	34.8	5.4	BL21, TB1	pMALc2X, pSUMO	IMAC, SEC, AC	1.7 × 10^4^ M^−1^s^−1^ 1.0 × 10^5^ M^−1^s^−1^	FK506 Rapamycin D44	320, 260 480 125	[111,112,116,159]
*P. vivax*	PvFKBP25	A0A1G4H4D0	25.2	9.5	BL21(DE3)	pNIC28-Bsa4	IMAC, SEC				[122]
	PvFKBP34 (PvFKBP35)	A0A565A3M9	34.0	6.1	BL21(DE3)	pSUMO	IMAC, SEC	1.0 × 10^5^ M^−1^s^−1^	FK506 D44	160 125	[122,159]
*G. intestinalis*	GiFKBP12	Q8I6M8	12.0	9.2	BL21(DE3)-R3-RARE	AVA0421	IMAC, SEC				[67]
*T. cruzi*	TcPar12.6 (TcPin1)	Q4D8F7/ Q4DKA4	12.6	7.7	JM109	pQE30	IMAC	3.97 × 10^5^ M^−1^s^−1^ 1.54 × 10^4^ M^−1^s^−1^			[82,85]
	TcPar13 (TcPar14)	Q4D394/ Q4E641	13.3	9.4	BL21(DE3)-CodonPlus RIL	pET-22b+	IMAC, SEC	0.194 M^−1^s^−1^			[83]
	TcPar45	Q4D9J4/ Q4DH56	45.5	8.7	BL21(DE3)-CodonPlus RIL	PET28a	IMAC, SEC	7.1 × 10^3^ M^−1^s^−1^			[83]
*T. brucei*	TbgPar12 (TbPin1)	C9ZUI9	12.5	6		pET28b	SEC				[147]
	TbPar42	C9ZRL7	41.7	7.1		pET28b	SEC				[147]
*L. major*	LmaPar13 (LmPIN1)	Q4QII4	12.6	7.2	BL21		IMAC, SEC				[100,101]

^1^ Experimental data reported in the cited references. ^2^ Previously reported PPIase names are presented in parentheses. ^3^ Reported catalytic efficiency values determined by Kofron assay. ^4^ Values for IC_50_. *Ki* only for LmaCyP19. ^5^ >200 nm for the four inhibitors. Blank spaces: no reported data. AC, affinity chromatography; IMAC, immobilized metal affinity chromatography; SEC, size exclusion chromatography; HIC, hydrophobic interaction chromatography; RPC, reversed-phase chromatography; IEX, ion-exchange chromatography; CsA, cyclosporin A; CsC, cyclosporin C; CsD, cyclosporin D (CsD); pI, isoelectric point; IC_50_, inhibitory concentration at 50%; *K_i_*, inhibitory constant.

## Supplementary Materials

The following supporting information can be downloaded at: https://www.mdpi.com/article/10.3390/pathogens13080644/s1, Figure S1: Phylogenetic tree of *T. vaginalis* PPIases compared to examples of PPIases from humans and *E. coli*; Figure S2: Sequence alignments of the CLD of *T. vaginalis* cyclophilins; Figure S3: Sequence alignment of the most similar members of the PPIAse CyP type and the 18 kDa CyP from *Entamoeba histolytica*; Table S1: Sequence identities of *T. vaginalis* PPIases; Table S2: Comparison of PPIases among *G. intestinalis* isolates and human orthologs; Table S3: Percent identity matrix for *G. intestinalis* PPIases; Table S4: Percentage identity among PPIase sequences of *L. major* and *L. donovani*; Table S5: Comparison of PPIase sequences between *P. falciparum* and *P. vivax*. Table S6. Identity percentages among PPIases of *C. parvum* and *C. hominis*.
