# Supplementary material for: Insights into Peptidyl-Prolyl cis-trans Isomerases from Clinically Important Protozoans: From Structure to Potential Biotechnological Applications
Source: Pathogens. 2024 Jul 31;13(8):644. doi: 10.3390/pathogens13080644 (PMC11357558; doi:10.3390/pathogens13080644)
Supplement: Supplementary file 1 [file pathogens-13-00644-s001.zip › pathogens-3075324-supplementary/Figure S1.pdf]

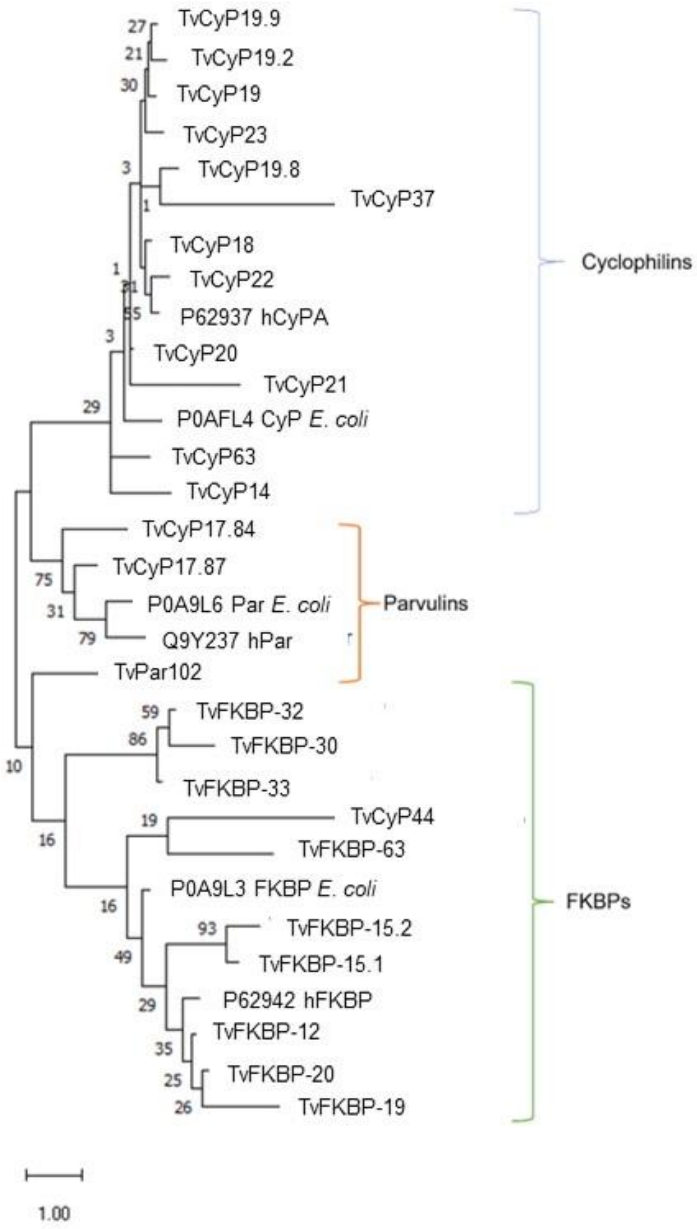

Figure S1. ***T. vaginalis* PPIases phylogenetic tree compared to examples of PPIases from Human and *E. coli*.** Brackets indicate cyclophilin families grouped together. Analysis made with MEGA 11.0[42] with a bootstrapping of 1000, using the Maximum Likelihood method and WAG+ G analysis.
