# Supplementary material for: Insights into Peptidyl-Prolyl cis-trans Isomerases from Clinically Important Protozoans: From Structure to Potential Biotechnological Applications
Source: Pathogens. 2024 Jul 31;13(8):644. doi: 10.3390/pathogens13080644 (PMC11357558; doi:10.3390/pathogens13080644)
Supplement: Supplementary file 1 [file pathogens-13-00644-s001.zip › pathogens-3075324-supplementary/Figure S3.pdf]

```
O15729      4 PKVFFDITIG.[2].KAGRIVMELFNDIVPKTAENFRCLCTGE.[ 6].YKGCGFHRVIKDFMIQGGDFTRHNGTGG 77  E. histolytica
AAQ94596   11 PIVFFDVSIG.[2].EVGRMKIELFADIVPKTAENFRQFCTGE.[ 9].YKGCTFHRVIKDFMIQGGDFVNGDGTGI 87  Zebrafish
NP_610224  17 PVVFFDIAVG.[2].EIGRMIFELFADTVPRTAENFRQFCTGE.[ 9].YKGASFHRVIKDFMIQGGDFVQGDGTGV 93  Fruit fly
B53522    179 PRVFFDISID.[2].PAGRIEFELFADVVPKTAENFRALCTGE.[11].YKGCPFHRIIPQFMCQGGDFTRMNGTGG 257 T. gondii
AAR11779    4 PRVFFDVTAN.[2].PVGRIMMELRGDVVPKTAENFRALCTGE.[ 4].YKGSPFHRVIPSFMCQGGDFTRQNGTGG 75  C. farreri
AAC05639    4 PIVFFDITAD.[2].PVGRIEMELYADDVPKTAENFRALCTGE.[11].FKGSTFHRVIPNFMCQGGDFTRGNGTGG 82  C. reinhardtii
EAA42921    7 DKVFFDITIG.[2].PLGRITMGLFGSIVPKTAENFKKLCTGE.[ 4].YKGSTFHRVIPKFMIQGGDFTNHNGTGG 78  G. lamblia
1AK4_A      4 PTVFFDIAVD.[2].PLGRVSFELFADKVPKTAENFRALSTGE.[ 4].YKGSCFHRIIPGFMCQGGDFTRHNGTGG 75  Human
CAE76450   12 PLVFFDITLG.[2].PLGRITFELFKDVVPRTAENFRQFCTGE.[10].YKGSKFHRIIPNFMCQGGDFLNGDGTGS 89  N. crassa

O15729     78      KSIYGTKFADEAFTVKH.[1].KPGMLSMANAGPNTNGSQFFITTVPCPWLDGKHVVFGQVVEGYDVVKMIENN 147 E. histolytica
AAQ94596   88      CSIYRGPFADENFRMKH.[1].GPGLLSMANSGPGTNGCQFFITCTKCDWLDGKHVVFGKVVDGLLVMRKIENV 157 Zebrafish
NP_610224  94      TSIYGNTFGDENFTLKH.[1].SPGLLSMANSGKETNGCQFFITCAKCNFLDGKHVVFGRVLDGLLIMRKIENV 163 Fruit fly
B53522    258      ESIYGEKFADENFSYKH.[1].EPFLLSMANAGPNTNGSQFFITTVPCPWLDGKHVVFGKVVAGQEVVKMMEAE 327 T. gondii
AAR11779   76      KSIYGEKFADENFTLKH.[1].GPGVLSMANAGPNTNGSQFFLCTARTEWLDGKHVVFGSVVEGMDVVKKIESY 145 C. farreri
AAC05639   83      ESIYGEKFPDENFKHRH.[1].GPGVLSMANAGPNTNGSQFFLCTVETAWLDGKHVVFGKVCKGMDVVKKVEGY 152 C. reinhardtii
EAA42921   79      KSIYGAKFPDENFEIKH     FVGCLSMANAGPNTNGSQFFLTVADTAWLDGKHVVFGRVLDGMDVVKAIETT 147 G. lamblia
1AK4_A     76      KSIYGEKFEDENFILKH.[1].GPGILSMANAGPNTNGSQFFICTAKTEWLDGKHVVFGKVKEGMNIVEAMERF 145 Human
CAE76450   90 .[1].CIYGTKSFADENFLLKH.[1].TPGLLSMANAGPNTNGSQFFITTVPTPFLDGKHVVFGKVVDGMDVVKKMENT 160 N. crassa

O15729    148 .[7].PKKAVVIADCG 165 E. histolytica
AAQ94596  158 .[7].PKLPIVVSQCG 175 Zebrafish
NP_610224 164 .[7].PKLPVTISQCG 181 Fruit fly
B53522    328 .[6].PKCAVEISSCG 344 T. gondii
AAR11779  146 .[6].TSEKIIVADCG 162 C. farreri
AAC05639  153 .[6].TRATFAIADCA 169 C. reinhardtii
EAA42921  148 .[7].PVEKVVIADCG 165 G. lamblia
1AK4_A    146 .[6].TSKKITIADCG 162 Human
CAE76450  161 .[9].PNLDVVIAQCG 180 N. crassa
```

Figure S3. Sequence alignment for the most similar members of the PPIAse CyP-type and the 18 kDa CyP from *Entamoeba histolytica*. All proteins are identified with NCBI Accession number, and the alignment was carried out in CDD data base [45]. Amino acids in the active site are in yellow boxes and conserved residues are in gray boxes.
