# Supplementary material for: Insights into Peptidyl-Prolyl cis-trans Isomerases from Clinically Important Protozoans: From Structure to Potential Biotechnological Applications
Source: Pathogens. 2024 Jul 31;13(8):644. doi: 10.3390/pathogens13080644 (PMC11357558; doi:10.3390/pathogens13080644)
Supplement: Supplementary file 1 [file pathogens-13-00644-s001.zip › pathogens-3075324-supplementary/Table S1.pdf]

Table S1. Sequence identities of *T. vaginalis* PPIases.

Reference PPIasa	PPIase	UniProt	Identity (%)
	TvCyP14	A2FJP1	19.0
	TvCyP18	A2EC21	71.7
	TvCyP19	A2DT06	100.0
	TvCyP19.2	A2F1H0	58.0
	TvCyP19.8	A2FAA8	44.0
**TvCyP19**	TvCyP19.9	A2DLL4	64.0
	TvCyP20	A2E5J4	53.7
	TvCyP21	A2FIV3	19.5
	TvCyP22	A2DKZ9	47.5
	TvCyP23	A2FTU8	56.7
	TvCyP37	A2E6H3	10.7
	TvCyP44	A2GDG2	11.0
	TvCyP63	A2DEW6	10.0
	TvFKBP12	A2DA37	100.0
	TvFKBP15.1	A2DYS7	19.4
	TvFKBP15.2	A2G763	16.7
**TvFKBP12**	TvFKBP19	A2FYT1	18.2
	TvFKBP20	A2F0D0	37.2
	TvFKBP30	A2EV02	24.8
	TvFKBP32	A2EC50	16.3
	TvFKBP33	A2G9L9	14.8
	TvFKBP63	A2FER9	6.6
	TvPar17.84	A2ECU0	100.0
**TvPar17.84**	TvPar17.87	A2ED59	41.6
	TvPar102	A2EWG2	5.3

Complete sequence alignment analysis of PPIases from *T. vaginalis* was run with the ClustalW2 tool embedded in EMBOSS-Needle [42]. CyPs were aligned with TvCyP19, FKBPs were aligned with TvFKBP-12, and Pars were aligned with TvPar17.84. The sequences were retrieved from the UniProt database [40] (https://www.uniprot.org/, Release 2023_02)
