# Supplementary material for: Insights into Peptidyl-Prolyl cis-trans Isomerases from Clinically Important Protozoans: From Structure to Potential Biotechnological Applications
Source: Pathogens. 2024 Jul 31;13(8):644. doi: 10.3390/pathogens13080644 (PMC11357558; doi:10.3390/pathogens13080644)
Supplement: Supplementary file 1 [file pathogens-13-00644-s001.zip › pathogens-3075324-supplementary/Table S2.pdf]

Table S2. Comparison of PPIases among *G. intestinal* isolates and human orthologues.

PPIasa	UniProt ID	*G. intestinalis* Isolate	Size		pI[3]	PPIase Domain	Additional regions	Human orthologue
			AAs	[2]kDa		(residues)	(residues)	
hCyP18[1] (CYPA)	P62937		165	18.01	7.68	7-163		
hCyP24[1] (CYPB)	P23284		216[3] 205[4]	23.74	9.42	47-204	SP (1–33) ER (213-216)	
GiCyP18	A8BC67	WB	168	18.04	8.42	10-166		hCyP18
GiCyP18	C6LQJ1	GS	168	18.03	8.42	10-166		hCyP18
GiCyP21	A8BJP8	WB	191	21.21	6.29	24-180	SP (1-13)	hCyP24
GiCyP21	C6LR04	GS	191	21.26	6.29	24-180	SP (1-13)	hCyP24
GiCyP25	V6TEN6	DH	230	25.49	6.33	63-219	TMH (31-51)	hCyP24
hFKBP12[1] (FKB1A)	P62942		108	11.95	7.89	20-108		
GiFKBP12	Q8I6M8	WB	109	11.85	9.22	22-109	DR (1-21)	hFKBP12
GiFKBP12	C6LUS9	GS	109	11.85	9.26	22-109	DR (1-21)	hFKBP12
GiFKBP13	A8B770	WB	111	12.56	9.52	22-110		
GiFKBP13	C6LPP4	GS	111	12.61	9.34	22-110		
GiFKBP24	A8BHU4	WB	215	23.85	6.30	127-215	SP (1-15)	
GiFKBP24	C6LXS7	GS	215	23.93	5.72	127-215	SP 1-15	
GiFKBP28	A8BUZ7	WB	244	27.80	4.68	128-225	SP (1-18)	
GiFKBP28	C6LY30	GS	244	27.87	4.65	128-225	SP (1-18)	
GiFKBP29	V6TL25	DH	251	28.55	4.65	135-232	SP (1-20)	
GiFKBP38	A8BAF3	WB	338	37.58	5.20	67-154	TPR(168-295)	FKB1A
GiFKBP38	C6LPE9	GS	338	37.68	5.20	67-154	TPR(168-295)	FKB1A
GiFKBP39	A8BK50	WB	354	38.87	6.44	265-354		FKB1A
GiFKBP39	C6M084	GS	356	39.02	6.35	267-356	DR(1-22, 217-243)	

[1] Human PPIase references. [2]Estimated Molecular weight (kDa) and Isoelectric point (pI) from PPIase sequence. WB: *Giardia* assemblage A isolate WB C6. DH: *Giardia* sub-assemblage A2 isolate DH. GS: *Giardia* assemblage B isolate GS/M clone H7 (GS).. [3] Precursor. [4]Mature protein. SP: Signal Peptide. ER: prevents secretion from Endoplasmic Reticulum motif. DR: Disorder Region. TMH: Trans Membrane Helical. TPR Tetratricopeptide repeat motif. All data deposited in the table were obtained from the UniProt database [40] (https://www.uniprot.org/, Release 2023_02).
