# Supplementary material for: Insights into Peptidyl-Prolyl cis-trans Isomerases from Clinically Important Protozoans: From Structure to Potential Biotechnological Applications
Source: Pathogens. 2024 Jul 31;13(8):644. doi: 10.3390/pathogens13080644 (PMC11357558; doi:10.3390/pathogens13080644)
Supplement: Supplementary file 1 [file pathogens-13-00644-s001.zip › pathogens-3075324-supplementary/Table S3.pdf]

Table S3. Percent identity matrix for *G. intestinalis* PPIases.

PPIase name	UniProt	Isolate	Percent Identity Matrix																				
GiCyP21	C6LR04	GS	100	94.24	94.24	51.31	47.56	54.17	54.17	16.09	16.09	16.09	16.33	13.61	23.81	22.62	15.97	14.58	15.71	15	25	20.25	20.25
GiCyP25	V6TEN6	DH	94.24	100	100	48.58	46.95	53.57	53.57	16.09	17.32	16.67	20.43	18.28	23.81	22.62	16.67	15.28	17.86	16.43	26.25	21.52	21.52
GiCyP21	A8BJP8	WB	94.24	100	100	50.79	46.95	53.57	53.57	16.09	16.67	16.67	17.69	14.97	23.81	22.62	16.67	15.28	17.86	16.43	26.25	21.52	21.52
hCYPS1	P23284PPIB	HUMAN	51.31	48.58	50.79	100	63.64	62.5	63.1	14.61	14.75	14.04	19.3	17.54	18.82	16.47	14.38	13.01	18.88	18.88	23.46	23.75	23.75
hCyPA	P62937|PPIA	HUMAN	47.56	46.95	46.95	63.64	100	66.46	65.85	12.99	12.34	12.34	14.62	13.85	16.67	14.29	12.59	12.59	13.28	11.72	18.75	18.75	18.75
GiCyP18	C6LQJ1	GS	54.17	53.57	53.57	62.5	66.46	100	99.4	14.01	13.38	13.38	15.04	13.53	17.86	15.48	14.81	14.81	15.5	13.95	20	20.25	20.25
GiCyP18	A8BC67	WB	54.17	53.57	53.57	63.1	65.85	99.4	100	14.01	13.38	13.38	15.79	14.29	17.86	15.48	14.81	14.81	14.73	13.95	20	20.25	20.25
GiFKBP28	C6LY30	GS	16.09	16.09	16.09	14.61	12.99	14.01	14.01	100	79.1	79.1	17.87	17.39	20	20	21.25	20.62	23.16	21.05	21.78	29	30
GiFKBP29	V6TL25	DH	16.09	17.32	16.67	14.75	12.34	13.38	13.38	79.1	100	100	14.95	14.95	21.9	20.95	18.12	17.5	21.58	19.47	19.8	27	27
GiFKBP28	A8BUZ7	WB	16.09	16.67	16.67	14.04	12.34	13.38	13.38	79.1	100	100	14.01	14.01	21.9	20.95	18.12	17.5	21.58	19.47	19.8	27	27
GiFKBP39	C6M084	GS	16.33	20.43	17.69	19.3	14.62	15.04	15.79	17.87	14.95	14.01	100	86.44	26.67	25.71	30.87	30.87	24.88	24.88	39.62	38.32	38.32
GiFKBP39	A8BK50	WB	13.61	18.28	14.97	17.54	13.85	13.53	14.29	17.39	14.95	14.01	86.44	100	25.71	26.67	28.86	28.86	25.94	25.47	38.68	37.38	37.38
GiFKBP13	C6LPP4	GS	23.81	23.81	23.81	18.82	16.67	17.86	17.86	20	21.9	21.9	26.67	25.71	100	91.89	30	29.09	33.02	33.96	37.38	38.68	39.62
GiFKBP13	A8B770	WB	22.62	22.62	22.62	16.47	14.29	15.48	15.48	20	20.95	20.95	25.71	26.67	91.89	100	30	29.09	35.85	34.91	36.45	38.68	39.62
GiFKBP38	C6LPE9	GS	15.97	16.67	16.67	14.38	12.59	14.81	14.81	21.25	18.12	18.12	30.87	28.86	30	30	100	94.97	32.67	31.33	48.6	48.15	48.15
GiFKBP38	A8BAF3	WB	14.58	15.28	15.28	13.01	12.59	14.81	14.81	20.62	17.5	17.5	30.87	28.86	29.09	29.09	94.97	100	32	31.33	48.6	47.22	47.22
GiFKBP24	C6LXS7	GS	15.71	17.86	17.86	18.88	13.28	15.5	14.73	23.16	21.58	21.58	24.88	25.94	33.02	35.85	32.67	32	100	84.19	42.06	47.22	48.15
GiFKBP24	A8BHU4	WB	15	16.43	16.43	18.88	11.72	13.95	13.95	21.05	19.47	19.47	24.88	25.47	33.96	34.91	31.33	31.33	84.19	100	41.12	47.22	48.15
FKBP-12	P62942|FKB1A	HUMAN	25	26.25	26.25	23.46	18.75	20	20	21.78	19.8	19.8	39.62	38.68	37.38	36.45	48.6	48.6	42.06	41.12	100	54.21	52.34
GiFKBP12	C6LUS9	GS	20.25	21.52	21.52	23.75	18.75	20.25	20.25	29	27	27	38.32	37.38	38.68	38.68	48.15	47.22	47.22	47.22	54.21	100	97.25
GiFKBP12	Q8I6M8	WB	20.25	21.52	21.52	23.75	18.75	20.25	20.25	30	27	27	38.32	37.38	39.62	39.62	48.15	47.22	48.15	48.15	52.34	97.25	100

GS: *Giardia* assemblage B isolate GS/M clone H7 (GS). DH: *Giardia* sub-assemblage A2 isolate DH. WB: *Giardia* assemblage A isolate WB C6. PPIA and PPIB are hCyPA and hCyPB, respectively. FKB1A is hFKBP-12. The Percent Identity Matrix was performed on the UniProt database [40] (https://www.uniprot.org/, Release 2023_02) and created using Clustal 2 [64].

100	99-95	94-90	89-80	79-70	69-60	59-50	49-40	39-30	29-20	<19	%
