# Supplementary material for: Insights into Peptidyl-Prolyl cis-trans Isomerases from Clinically Important Protozoans: From Structure to Potential Biotechnological Applications
Source: Pathogens. 2024 Jul 31;13(8):644. doi: 10.3390/pathogens13080644 (PMC11357558; doi:10.3390/pathogens13080644)
Supplement: Supplementary file 1 [file pathogens-13-00644-s001.zip › pathogens-3075324-supplementary/Table S4new.pdf]

Table S4. Comparison of PPIase sequences between *L. major and L. donovani.*

L. major					*L. donovani*					Identity (%)
Name	Length (aa)	kDa	PPIase domain Region	PPIase domain (aa)	Name	Length (aa)	kDa	PPIase domain Region	PPIase domain (aa)	
LmCyP19	177	18.8	17-176	159	LdCyP19	177	18.8	17-176	159	96
LmCyP20.3	187	20.3	30-186	156	LdCyP20.3	192	20.4	4-156	152	88.5
LmCyP20.4	192	23	18-156	138	LdCyP20.4	187	20.34	30-186	156	82.9
LmCyP24	220	24	50-216	166	LdCyP24	229	24.5	64-223	159	83.3
LmCyP24.6	229	24.6	64-223	159	LdCyP25	229	25.2	30-194	164	98.7
LmCyP25	229	25.2	30-194	164	LdCyP26	247	25.5	1-160	160	98.7
LmCyP26	247	25.5	1-160	160	LdCyP27	242	26.6	71-242	171	93.9
LmCyP26.5	242	26.4	71-242	171	LdCyP28	245	27.5	56-216	160	93
LmCyP27	245	27.5	56-216	160	LdCyP28.6	258	28.6	88-254	166	98.4
LmCyP29	266	28.8	83-265	182	LdCyP29	266	28.8	83-265	182	93.2
LmCyP32	295	31.4	27-193	166	LdCyP32	296	31.9	27-193	166	91.2
LaCyP36	335	36.1	153-330	177	LdCyP36	334	36.09	152-329	177	93.7
LmCyP38	354	38.4	7-174	167	LdCyP38.4	354	38.4	7-174	167	96
LmCyP39	366	38.6	80-243	163	LdCyP39	366	38.7	80-243	163	94
LmCyP48	444	48.5	222-376	154	LdCyP48.5	444	48.5	222-376	154	94
LmCyP49	462	49	58-238	180	LdCyP49	461	48.8	59-239	180	87.4
LmCyP108	1020	108.1	863-1017	154	LdCyP108	1022	108.3	865-1019	154	93.8
LmFKBP-11.8	111	11.8	23-111	88	LdFKBP-11.8	111	11.8	23-111	88	98.2
LmFKBP-11.9	109	11.9	19-109	90	LdFKBP-11.9	109	11.9	19-109	90	96.3
LmFKBP-17.3	159	17.2	49-135	86	LdFKBP-17	159	17.3	49-135	86	93.7
LmFKBP-23	202	22.8	81-168	87	LdFKBP-22	201	22.7	80-167	87	96.5
LmFKBP-48	432	47.6	56-144	88	LdFKBP-47	432	47.8	56-144	88	96.1
LmPar13	115	12.5	3-115	112	LdPar12	115	12.6	3-115	112	93.9
LmPar47	440	46.5	313-440	127	LdPar17	160	17.4	33-160	127	34.3

The numbers in each region indicate the starting and ending amino acid position of the PPIase domain. The percentage of identity was obtained by comparing the amino acid sequences of *L. major* PPIases with the *L. donovani* PPIases using the EMBOSS Needle tool from EMBL-EBI [42] (https://www.ebi.ac.uk/services). The molecular weight (kDa) and amino acids sequence were obtained from the UniProt database [40] (https://www.uniprot.org/, Release 2023_02).
