# Supplementary material for: Insights into Peptidyl-Prolyl cis-trans Isomerases from Clinically Important Protozoans: From Structure to Potential Biotechnological Applications
Source: Pathogens. 2024 Jul 31;13(8):644. doi: 10.3390/pathogens13080644 (PMC11357558; doi:10.3390/pathogens13080644)
Supplement: Supplementary file 1 [file pathogens-13-00644-s001.zip › pathogens-3075324-supplementary/Table S5new.pdf]

Table S5. Comparison of PPIase sequences between *P. falciparum* and *P. vivax*.

P. falciparum					***P. vivax***					**Identity (%)**
**Name**	**Length** (aa)	**kDa**	**PPIase domain** Region	(aa)	**Name**	**Length (aa)**	**kDa**	**PPIase domain** Region	(aa)	
PfCyP18.6	167	18.6	6-154	148	PvCyP18.4	167	18.4	6-154	148	88.6
PfCyP19	171	19	8-170	162	PvCyP19	170	19	7-169	162	88.9
PfCyP22	195	22	32-192	160	PvCyP21	193	21	32-192	160	79.0
PfCyP23	204	23	53-199	146	PvCyP23	203	23	52-198	146	85.3
PfCyP25	217	24.9	51-216	165	PvCyP29	262	29	96-261	165	66.8
PfCyP26	226	26	9-173	162	PvCyP26	226	26	9-173	162	87.6
PfCyP32	280	32	65-223	158	PvCyP32	276	32	65-223	158	80.0
PfCyP53	446	52.6	19-161	142	PvCyP52	456	52	19-160	141	55.4
PfCyP72	609	72.5	333-526	193	PvCyP71	616	71	322-508	186	59.0
PfCyP81	677	81	8-217	209	PvCyP65	590	65	8-194	186	37.8
PfCyP87	747	87	599-745	146	PvCyP83	737	83	589-735	146	66.4
PfFKBP-25	213	25.6	100-185	85	PvFKBP-25	215	25	102-187	85	60.0
PfFKBP-35	304	35	37-126	89	PvFKBP-34	302	34	36-125	89	79.7

The numbers in each region indicate the starting and ending amino acid position of the PPIase domain. The percentage of identity and similarity were obtained by comparing the amino acid sequences of *P. falciparum* PPIases with the *P. vivax* PPIases using the EMBOSS Needle tool from EMBL-EBI (https://www.ebi.ac.uk/services) [42]. Proteins, PfCyP22 and PvCyP21, were considered precursors, i.e., uncleaved signal peptides. All data deposited in the table were obtained from the UniProt database [40] (https://www.uniprot.org/, Release 2023_02).
