# Supplementary material for: Insights into Peptidyl-Prolyl cis-trans Isomerases from Clinically Important Protozoans: From Structure to Potential Biotechnological Applications
Source: Pathogens. 2024 Jul 31;13(8):644. doi: 10.3390/pathogens13080644 (PMC11357558; doi:10.3390/pathogens13080644)
Supplement: Supplementary file 1 [file pathogens-13-00644-s001.zip › pathogens-3075324-supplementary/Table S6new.pdf]

Table S6. Comparison of PPIase sequences between *C. parvum* and *C. hominis*.

C. parvum					*C. hominis*					Identity (%)
Name	Length (aa)	kDa	PPIase domain Region	(aa)	Name	Length (aa)	kDa	PPIase domain Region	aa	
CpCyP18	172	18.5	7-170	163	ChCyP18	172	18.5	7-170	163	100
CpCyP19	169	18.8	7-157	150	ChCyP19	169	18.8	7-157	150	98.8
CpCyP21.1	198	21.1	34-197	163	ChCyP21	198	21.0	34-197	163	98.5
CpCyP21.2	189	21.2	28-188	160	ChCyP21.2	189	21.2	28-188	160	98.9
CpCyP23	210	22.8	42-198	156	ChCyP23	210	22.9	42-198	156	98.6
CpCyP34	302	34.5	6-162	156	ChCyP40	342	39.6	6-162	156	87.4
CpCyP89	778	89	621-775	154	ChCyP89	774	88.8	617-771	154	96.9
CpFKBP-34	312	33.9	227-311	84	ChFKBP-34	312	33.9	227-311	84	96.5
CpFKBP-37	325	36.8	220-307	87	ChFKBP-37	325	36.8	220-307	87	98.8

The molecular weight (kDa) and amino acids sequence were obtained from the UniProt database [40] (https://www.uniprot.org/, Release 2023_02). Identities were obtained using the EMBOSS Needle tool from EMBL-EBI [42] (https://www.ebi.ac.uk/services).
