# Supplementary material for: Insights into Peptidyl-Prolyl cis-trans Isomerases from Clinically Important Protozoans: From Structure to Potential Biotechnological Applications
Source: Pathogens. 2024 Jul 31;13(8):644. doi: 10.3390/pathogens13080644 (PMC11357558; doi:10.3390/pathogens13080644)
Supplement: Supplementary file 1 [file Figure S2.pdf › pathogens-3075324-supplementary/Figure S2.pdf]

```
 TVAG_106810_Cyp        FVVDLFLKTAPMQGFNMLKLAKINY-----------FNNSLVLEIMKDFYLRIGNLQNPG    60
 TVAG_583670_Cyp        VILELDSDKTPMGVLNFLGHALRGS-----------YDKAEVKKVDQGKFLEIA----SS    287
 TVAG_078570_Cyp        VIFGLYYNECPKTVENYLAAFSCQP------KREFCYNNTYVAKLNPNKRLSIGVTLATK    87
 P0AFL4_Ecoli_Cyp       IELELDKQKAPVSVQNFVDYVNSGF-----------YNNTTFHRVIPGFMIQGGGFTEQM    88
 TVAG_047830_Cyp        VKIELRPDICPLACENFRRFCTGEYR---PKGVPLGYKGTRFHRVVPGFIVQGGDCDTQD    83
 TVAG_038810_Cyp        IHIGLFGNDVNKTAENFLHLCLCDQGIGT-QGKPLCYKGSPFHRIIPGFMIQGGDFTHQS    94
 TVAG_027250_Cyp        IVIELFVDRVPKTAENFRMLCTGEKGVGR-SGAKLHYKGCKFHRIIPHFLIQSGDFISND    82
 TVAG_146960_Cyp        IVMELRTDVCPKTCTNFLMLCTGQRGNSKISRKPLHYKGCPFHRIIPGFMAQGGDFTMGN    81
 TVAG_277390_Cyp        IIFELFSDIVPKTSENFRCLCTGEKGKGK-NGMPLHYKGTQFHRIIPNFMIQGGDIINFN    109
 P62937_Hum_Cyp_A       VSFELFADKVPKTAENFRALSTGEKGFG--------YKGSCFHRIIPGFMCQGGDFTRHN    71
 TVAG_062520_Cyp        IKFGLFGDDVPKTAENFRALCTGEKGMGK-LGKPLHYKGSPFHRVIPNFMIQGGDITSGN    91
→TVAG_004440_Cyp        IVFELFNDIVPKTAENFRALCTGEKGIGK-SGMPLSYKGTMFHRIIPQFMIQGGDFTRFN    79
 TVAG_137880_Cyp        IVFELYSDIVPKTAENFRCLCTGEKGTGK-SGKPLHYKGCAFHRIIPQFMIQGGDFTLGN    79
 TVAG_172150_Cyp        IKFEMFPEECPLTVENFVTHSKRGY-----------YDNTRIFRVERDFCIQMGDPT-GS    454
 TVAG_370440_Cyp        ------------------MLCASGY-----------YDHCPIHRVIQNYLVQTGDPT-FT    30
                                                            :     .:
                                                            Y     RI

 TVAG_106810_Cyp        ---GTTVYQLLTNNNTMYIRDEINNKL---SHSTAGFISTSN-SAKDQNTSEFFITLGRD    113
 TVAG_583670_Cyp        AISDESVWGTPFS--------FERNEPRRGMRYQIYIVKKNKNISRISNTADFGISKE--    337
 TVAG_078570_Cyp        -----SVKGDHESN------HEENK-L---LHDRAYLIT-SNTHYKTFLTGEFYITFRAW    131
 P0AFL4_Ecoli_Cyp       QQKKPNPPIKNEA--------DNGL------RNTRGTIAMARTADKDSATSQFFINVADN    134
 TVAG_047830_Cyp        GYGCISIYGREFN--------DEPSAY---KFDETGIVAMAN-SGPNSNGCQFFITMNPA    131
 TVAG_038810_Cyp        GVGGESIYGRHFD--------DENFKV---EHE-VGVISMAN-AGPNTNGSQFFITVAAT    141
 TVAG_027250_Cyp        GTSNESVYGQRFE--------DENFSV---KHDQPYIVSMAN-SGRNTNGGQFFITLVKA    130
 TVAG_146960_Cyp        GKGGESIYGQYFD--------DENFEL---KHEGPGVLSMAN-SGPNKNRSQFFICFKAQ    129
 TVAG_277390_Cyp        GTGGESIYGYTFP--------DENFYV---KHDNPYILSMAN-AGQNTNSSQFFITTVKT    157
 P62937_Hum_Cyp_A       GTGGKSIYGEKFE--------DENFIL---KHTGPGILSMAN-AGPNTNGSQFFICTAKT    119
 TVAG_062520_Cyp        GYGGESIYGSKFA--------DESFKI---THDGPGLLSMAN-SGPNTNGSQFFITTVPC    139
→TVAG_004440_Cyp        GTGGESIYGMKFD--------DENFKV---KHDKPGLLSMAN-AGPNTNGSQFFITTVET    127
 TVAG_137880_Cyp        GCGGESIYGAKFA--------DENFKA---KHTRPGLLSMAN-AGPNTNGSQFFITTVPC    127
 TVAG_172150_Cyp        GIGGESIWGGYFD--------DENLDNVINNFSEAWMVGMAN-EGKNTNGSQFFITTNPA    505
 TVAG_370440_Cyp        GDGGASIYGEKVQ--------VEPFGD----FSEPGYL--AY-AETHEVGSQFFITAAST    75
                                                             :             :* *
                                                             L             QF I

 TVAG_106810_Cyp        LSRFDS-----IRTIFGRISENFE-LIKSISEEFV----DETYRPYKNIRILGTKILYDP    163
 TVAG_583670_Cyp        P-LSID-----QYHIIGCVKSGEGYVASIIDGKT-----LPNGRPVRPCTIYNVEV---Q    383
 TVAG_078570_Cyp        P-KYNE-----ENIVFGRVLRGFD-VLEKLKIN-------SNEKDVDKIKIIDSGIIQDD    177
 P0AFL4_Ecoli_Cyp       A-FLDHGQRDFGYAVFGKVVKGMD-VADKISQVPTHDVGPYQNVPSKPVVILSAKVLP--    190
 TVAG_047830_Cyp        P-ELND-----SYVVIGKVFEGML-VLRHIESVPLK---PHSEIPTLDCIISQCGEL---    178
 TVAG_038810_Cyp        N-WLNG-----HHVVFGRVVKGMD-VVKKIEKVG-----SSSGTPSKKVVIVDTGLVE--    187
 TVAG_027250_Cyp        P-WLDF-----RHVAFGKVIEGTD-IVELIQAAG-----SSNGAPKQTILISDCGEI---    175
 TVAG_146960_Cyp        P-SLDG-----KHVVFGKVVEGFDEVVLQLEKLG-----SKKGETSKQILIADCGVLDAN    178
 TVAG_277390_Cyp        P-QLDG-----EHVAFGRVVNGFD-VVEKIQECR-----SDDGKPKFVCEVADCGQFD--    203
 P62937_Hum_Cyp_A       E-WLDG-----KHVVFGKVKEGMN-IVEAMERFG-----SRNGKTSKKITIADCGQLE--    165
 TVAG_062520_Cyp        P-WLNG-----KHVVFGKVIEGME-IVKKIESLG-----SQSGTPKAKIIIADCGEITE-    186
→TVAG_004440_Cyp        P-WLDG-----HHCVFGQVIEGMD-IVKQIESCG-----TESGRPRAMCMVTDCGEMK--    173
 TVAG_137880_Cyp        P-WLDG-----KHCVFGEVVEGMD-VVKALEKCG-----SDQGKTSKKCVITDCGQL---    172
 TVAG_172150_Cyp        P-SLNG-----KHTCWGRLVSGKETIQK-IMTVEID----KYKHPLDPILIVNI---TFS    551
 TVAG_370440_Cyp        P-ELAG-----EYTAFGHVIYGLNNIIA-FSRMPTF----EDHQPIRPAAIKSI---TIH    121
                                        * :      :   :                    :
                                        G V      I   I                    I
```

Figure S2. Sequence alignments of the CLD of *T. vaginalis* cyclophilins. Alignments were run with ClustalW2 tool embedded in EMBOSS-Needle[42]. Amino acids of the catalytic site in CLD in pink, conserved amino acids of CyPs from *T. vaginalis* in green. "**:**" Indicates strong groups fully conserved, "*****" Indicates a single fully conserved residue. Arrows indicate TvCyP19 sequence. Sequences were retrieved from UniProt database[25] (https://www.uniprot.org/, Release 2023_02).
